# Quantitative zeptomolar imaging of miRNA cancer markers with nanoparticle assemblies

**DOI:** 10.1073/pnas.1810764116

**Published:** 2019-02-11

**Authors:** Aihua Qu, Maozhong Sun, Liguang Xu, Changlong Hao, Xiaoling Wu, Chuanlai Xu, Nicholas A. Kotov, Hua Kuang

**Affiliations:** ^a^State Key Lab of Food Science and Technology, School of Food Science and Technology, Jiangnan University, Wuxi, Jiangsu 214122, China;; ^b^International Joint Research Laboratory for Biointerface and Biodetection, Jiangnan University, Wuxi, Jiangsu 214122, China;; ^c^Department of Chemical Engineering, Biointerface Institute, University of Michigan, Ann Arbor, MI 48109;; ^d^Michigan Institute of Translational Nanotechnology, Ypsilanti, MI 48198

**Keywords:** assembly, nanoparticles, miRNA, superstructures, cancer

## Abstract

Multiplexed detection of small noncoding RNAs responsible for posttranscriptional regulation of gene expression, known as miRNAs, is essential for understanding and controlling cell development. However, the lifetimes of miRNAs are short and their concentrations are low, which inhibits the development of miRNA-based methods, diagnostics, and treatment of many diseases. Here we show that DNA-bridged assemblies of gold nanorods with upconverting nanoparticles can simultaneously quantify two miRNA cancer markers, namely miR-21 and miR-200b. Energy upconversion in nanoparticles affords efficient excitation of fluorescent dyes via energy transfer in the superstructures with core–satellite geometry where gold nanorods are surrounded by upconverting nanoparticles. Spectral separation of the excitation beam and dye emission wavelengths enables drastic reduction of signal-to-noise ratio and the limit of detection to 3.2 zmol/ng_RNA_ (0.11 amol or 6.5 × 10^4^ copies) and 10.3 zmol/ng_RNA_ (0.34 amol or 2.1 × 10^5^ copies) for miR-21 and miR-200b, respectively. Zeptomolar sensitivity and analytical linearity with respect to miRNA concentration affords multiplexed detection and imaging of these markers, both in living cells and in vivo assays. These findings create a pathway for the creation of an miRNA toolbox for quantitative epigenetics and digital personalized medicine.

## Significance

Engineering of reconfigurable nanoparticle assemblies enabled the practical realization of multiplexed detection, monitoring, and in vivo imaging of miRNA in live cells and animals, which was previously impeded by the fast degradation of these essential epigenetic markers. Nanoparticle superstructures afford rapid quantitative assessment of the imaging results, facilitating proliferation of digital personalized medicine.

Relatively short segments of ribonucleic acids containing about 20 bases known as miRNAs represent the key gene expression regulators (1, 2). Accurate enumeration of miRNA concentrations inside and outside of cells is essential for deciphering cellular signaling pathways, understanding cellular epigenetics, and predicting the malignant progression of tumors (3–5). Adequate and consistent digitation of miRNA levels would also be essential for multiparameter diagnostics as a part of big-data toolbox of biomedicine. Real-time quantification of intracellular and extracellular miRNAs has been, so far, difficult because the concentration of these biomolecules per cell is in the attomolar range or even lower (6–10). Affinity microarrays and real-time qPCR techniques currently being used for quantification of DNA unfortunately have limited utility for miRNA because of rapid degradation of these biomolecules in the course of bioanalysis, for example PCR cycling (11, 12).

Imaging techniques using miRNA contrast agents would be highly desired as well but their development is equally difficult for the same chemical reasons (13–17). The early implementations of miRNA imaging was based on FRET or, more generally, luminescence resonance energy transfer (LRET) as well as on surface-enhanced Raman scattering (18–21). Improvements in miRNA analysis taking advantage of LRET involve optimization of the spectral characteristics of energy donors and acceptors. On one hand, their absorption and emission spectra should overlap for the energy transfer to occur (22). On the other hand, the spectral characteristics should allow for their independent quantification. LRET biosensing also imposes restrictions on the excitation spectra of the donor and acceptor. Their minimal overlap reduces the signal-to-noise ratio and improves the sensitivity of the analysis and the limit of detection (LOD) but is difficult to realize in concert with emission requirements. Finally, the linker between energy excitation donor and acceptor should be short enough to afford resonance between their excited states, yet long enough to have specificity for target biomolecules (23, 24). The significance of the linker and, more generally, the geometrical design of LRET systems for biosensing have been demonstrated for a variety of nanoparticle (NP) assemblies and other superstructures (25–30) used for both detection and bioimaging (31–35).

Optimization of all these conflicting requirements for miRNA detection is possible using DNA-bridged assemblies of upconverting NPs (UCNPs), gold nanorods (AuNR), and dye molecules (TAMRA and Cy5.5). UCNPs can be an attractive platform for LRET biosensing because they afford wide separation of excitation bands while providing sufficient overlap of the emission bands as well as photostability (22, 36–43). The length of miRNA was shown to be sufficient for their successful identification using chiroptical properties of UCNP assemblies (6–9, 44, 45).

In this study, we demonstrate that AuNR–UCNP assemblies of NPs can be designed to afford LRET-based detection of two miRNAs with zeptomolar LOD. These analytical capabilities and the resolution of the conundrum of spectroscopic requirements becomes possible when these superstructures have core–satellite geometries conducive to strong resonance between excited states of NPs, nanorods, and dye molecules (7, 46, 47). The exceptionally low LOD, high sensitivity, extended linearity, and high specificity of the emission intensities of TAMRA and Cy5.5 dyes excited by the upconverted photons from UCNPs eliminated cross-talk between luminescence signals and made possible quantitative miRNA imaging in cells and live animals with zeptomolar sensitivity.

## Results and Discussion

### Engineering of NP Assemblies for Multiplexed Quantification of miRNA.

**Fig. 1.**
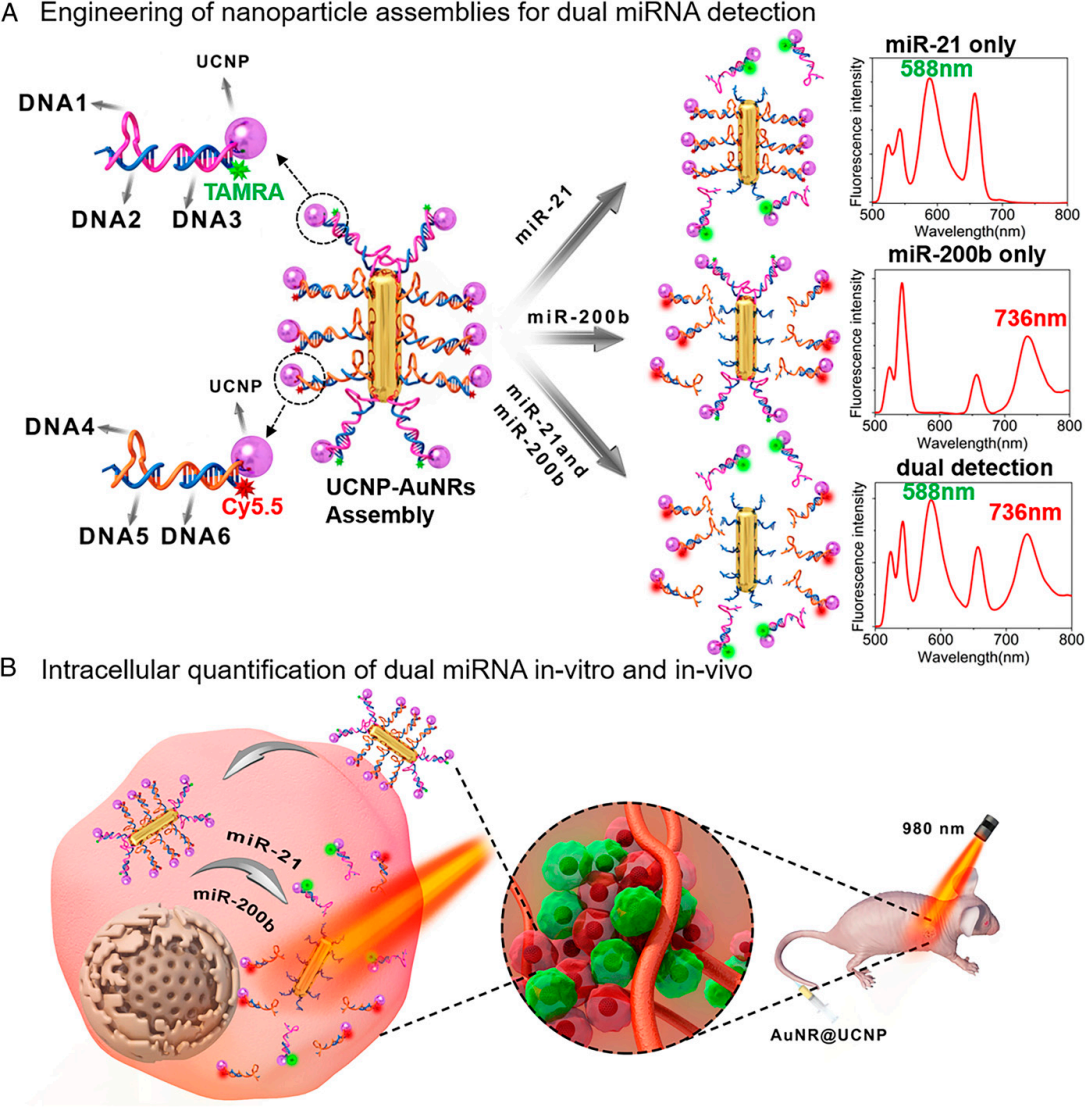
Schematic illustration of AuNR@UCNP core–satellite assemblies used for the simultaneous analysis of intracellular miR-21– and miR-200b–based LRET. (*A*) The fabrication routes for AuNR@UCNP core–satellite assemblies. (*B*) The AuNR@UCNP core–satellite assemblies used for dual target detection and in vivo images.

AuNR–UCNP assemblies (AuNR@UCNP) were designed in this work according to Fig. 1. Their core–satellite geometries were constructed using the principles of DNA origami based on previous studies in this area (48–51), taking advantage of six nucleic acid sequences given in *SI Appendix*, Table S1. To assemble the components, the ends of the AuNRs were modified with 5′-thiolated DNA_2_, and the sides of the AuNRs were functionalized with 5′-thiolated DNA_5_ in hexadecyltrimethylammonium bromide–Tris buffer. The controllable modification of single-stranded thiolated DNA on the sides or ends of the AuNRs was crucial for this work (31). The miRNA recognition sequences were embedded in DNA1 (miR-21, red-violet color in Fig. 1*A*) and DNA4 (miR-200b, orange color in Fig. 1*A*) and were linked to the UCNPs. TAMRA-modified DNA_3_ and Cy5.5-modified DNA6 were then connected to DNA1 and DNA4, respectively. Finally, the AuNRs and UCNPs were combined to form the complete core–satellite assembly with the hybridization of the complementary DNAs: DNA1 to DNA2, and DNA4 to DNA5.

To ensure that the assembly was uniformly dispersed in the biological environment, thiolated poly(ethylene) glycol (molecular weight 5,000) was reacted with the AuNRs and UCNPs to form a stable protective layer on them. Cell-penetrating peptide (TAT) was also added to the coating on the assembly to improve the efficiency of cell membrane penetration.

The UCNP luminescence of core–satellite superstructure (540 nm and 660 nm) was largely quenched due to the LRET to AuNRs under 980-nm excitation after assembly. When miR-21 was present, the recognition sequence in DNA1 linked to the end of AuNR, hybridized to the miRNA target. These binding events led to the dissociation of UCNPs from the ends of the AuNR, and the UCL was recovered. The overlap between the UCNP emission band at 540 nm and the excitation band of TAMRA at 559 nm affords the energy transfer from the dissociated UCNP to the fluorescent dye, under 980-nm excitation, which led to the lighting up of TAMRA dye (588 nm) in the presence of miR-200b, which recognizes the sequence of DNA4. Then, the UCNPs on the sides of AuNRs dissociated, resulting in the excitation of Cy5.5 (736 nm) fluorescence by dissociated UCNP. When both miR-21 and miR-200b are present, the dispersions simultaneously display emission of TAMRA (588 nm) and Cy5.5 (736 nm). Therefore, according to the intensity of two dyes excited by 980 nm, two miRNAs could be detected respectively or simultaneously, allowing the independent quantification of these cancer markers with one superstructure (Fig. 1*A*). Fig. 1*B* is a schematic illustration of the AuNR@UCNP core–satellite assembly used for the simultaneous analysis of the expression levels of miR-21 and miR-200b. After tail vein injection of assembly, the emission intensities of TAMRA and Cy5.5, respectively, in HeLa cell cultures and at the tumor sites of mice were imaged under 980-nm excitation, which could qualitatively detect the miRNA level in vivo.

### Structural Characterization and Optical Properties of AuNR@UCNP Assemblies.

**Fig. 2.**
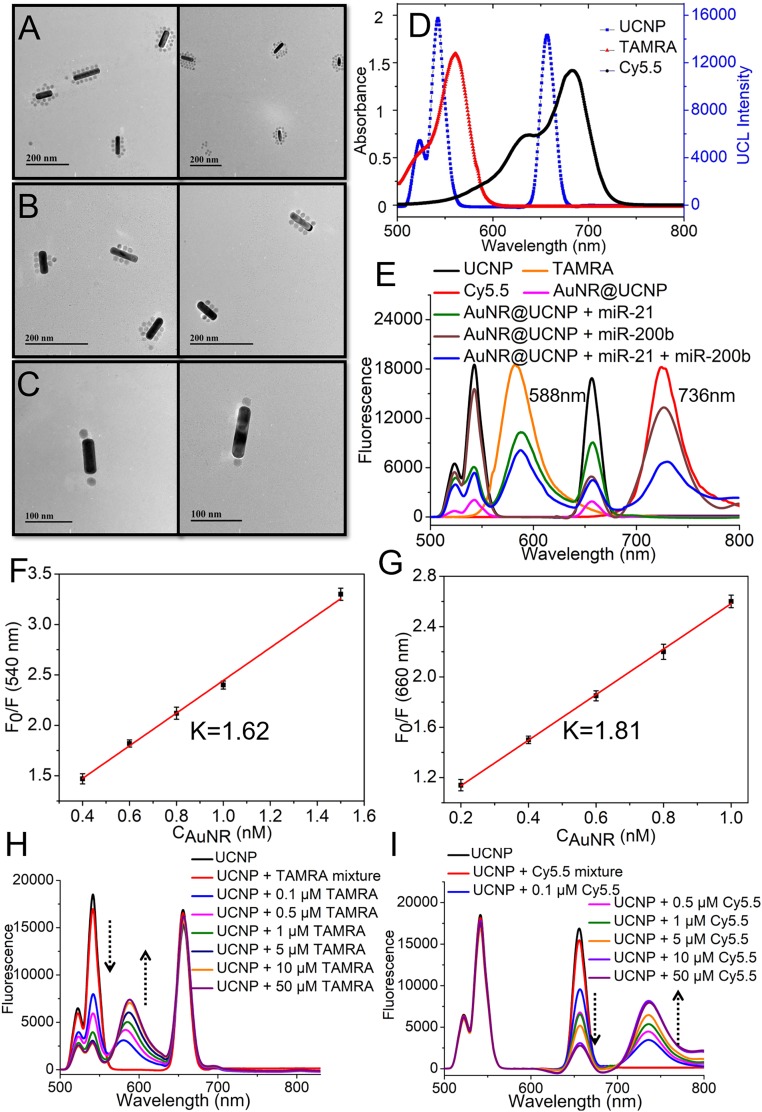
Characterization of AuNR@UCNP core–satellite assemblies. TEM images of (*A*) AuNR@UCNP assemblies, (*B*) AuNR@UCNP assembly with miR-21, and (*C*) AuNR@UCNP assemblies with miR-200b. (*D*) UV-Vis absorption and fluorescence spectra of UCNP, Cy5.5, and TAMRA. (*E*) Fluorescence spectra of UCNP, Cy5.5, TAMRA, AuNR@UCNP, AuNR@UCNP with miR-21, AuNR@UCNP with miR-200b, and AuNR@UCNP with miR-21 and miR-200b. The fluorescence was excited by illumination with photons with 980-nm wavelength and the power is 500 mW. (*F* and *G*) Emission intensity at 540 nm and 660 nm vs. the concentration of AuNRs. F_o_ and F represent the fluorescent intensity of UCNPs in the absence and presence of AuNRs, respectively. The concentration of UCNPs is 0.1 mg/mL. (*H*) Emission intensity of UCNP, UCNP, and TAMRA mixture, UCNP assembled with different concentrations of TAMRA modified DNA under 980-nm excitation and the power is 500 mW. (*I*) Emission intensity of UCNP, UCNP, and Cy5.5 mixture, UCNP assembled with different concentrations of Cy5.5 modified DNA under 980-nm excitation and the power is 500 mW. Error bars in *F* and *G* are mean ± SD (*n* = 3 independent samples).

AuNRs (60 ± 5 nm; *SI Appendix*, Fig. S1*A*) and UCNPs (20 ± 3 nm; *SI Appendix*, Fig. S1*B*) were assembled on a DNA frame according to the protocol given in *Materials and Methods* (*SI Appendix*, Table S1). The average length and width of the AuNR was about 60 ± 5 nm and 10 ± 3 nm, respectively, and the aspect ratio was about 6:1. Transmission electron microscopy (TEM) images showed the successful preparation and dispersion of the AuNR@UCNP core–satellite assemblies (Fig. 2*A*). The average number of UCNPs assembled on the AuNRs was 13 ± 4 (*SI Appendix*, Fig. S2). From the results of dynamic light scattering, the average hydrodynamic diameter of the AuNR@UCNP assemblies was 143 ± 6 nm, which is much larger than those of individual AuNRs (70 ± 5 nm) and UCNPs (27 ± 3 nm) (*SI Appendix*, Fig. S3), confirming their successful assembly. The UV-visible (UV-Vis) spectra displayed a bathochromic shift of the AuNR absorbance band, from 742 to 751 nm (*SI Appendix*, Fig. S4), which further substantiated the conclusion.

Individual UCNPs showed strong luminescence at 540 nm and 660 nm. If the UCNPs were assembled with the AuNRs in addition to dyes, the light emission from all states was quenched due to energy transfer to the plasmonic states of nanorods and their fast thermalization (Fig. 2*E* and *SI Appendix*, Fig. S4). The efficiency of quenching was tested by varying the concentration of AuNRs, while maintaining a constant UCNP concentration. The results showed that the upconversion luminescense (UCL) intensity decreased as the amount of AuNRs increased (*SI Appendix*, Fig. S5), and the degree of UCL quenching was higher at 660-nm emission (1.81) than at 540-nm emission (1.62) (Fig. 2 *F* and *G* and *SI Appendix*, Figs. S6 and S7) (16, 52). With UCNP acting as energy donors, TAMRA and Cy5.5 were chosen as energy acceptors. The emission peaks of the UCNPs at 540 nm and 660 nm overlap the absorbance peaks of TAMRA (559 nm) and Cy5.5 (685 nm), respectively (Fig. 2*D*). Thus, emission of TAMRA and Cy5.5 at 559 and 685 nm, respectively, was observed upon excitation of UCNPs at 980 nm, indicating excitation transfer from UCNPs to dyes. Fullfilling the requirements for of LRET bisensor design, the intensity of the donor emission in the presence of the target miRNAs was low, suggesting that the probe had high energy transfer efficiency.

We also measured the luminescence intensity of UCNPs assembled with different concentrations of TAMRA-modified DNA (Fig. 2*H*). The results showed that when the concentration of TAMRA-modified DNA increased the UCL intensity at 540 nm decreased. Concomitantly, the characteristic TAMRA emission intensity at 588 nm was gradually enhanced at 980-nm excitation (Fig. 2*H*), whereas UCL intensity at 660 nm remained unchanged. Also, when the UCNPs were assembled with increased concentration of Cy5.5-modified DNA, the UCL intensity at 660 nm decreased while the characteristc Cy5.5 intensity at 736 nm gradually increased (Fig. 2*I*). The critical role of the assemblies for bioanalytical purposes can be demonstrated by the fact that the mixing of “free” UCNP with the TAMRA or Cy5.5 leads to no change in UCL from the NPs.

When UCNPs encountered target miRNAs they disassembled from AuNR, allowing the energy transfer between UCNPs and dye molecules to occur (Fig. 2*E*). When in the presence of only miR-21, the UCNPs attached to the ends of the AuNRs dissociated from them (Fig. 2*B*), together with TAMRA-modified DNA_3_. The UCL was restored, which led to the excitation of TAMRA fluorescence at 540 nm through LRET from UCNP when illuminated under 980 nm. However, the Cy5.5 could not be excited because the Cy5.5-modified DNA_6_ was undissociated from AuNR (Fig. 2*E*). In the presence of miR-200b, the UCNPs at the sides of the AuNRs were dissociated (Fig. 2*C*), which resulted in high intensity of Cy5.5 fluorescence at 736 nm through LRET of UCNP at 660 nm, while the TAMRA was quenched. In the presence of both miR-21 and miR-200b, TAMRA and Cy5.5 both displayed strong fluorescence, while the emission of UCNPs was quenched (Fig. 2*E*).

### Detection of miR-21 and miR-200b by AuNR@UCNP Assemblies in Vitro.

**Fig. 3.**
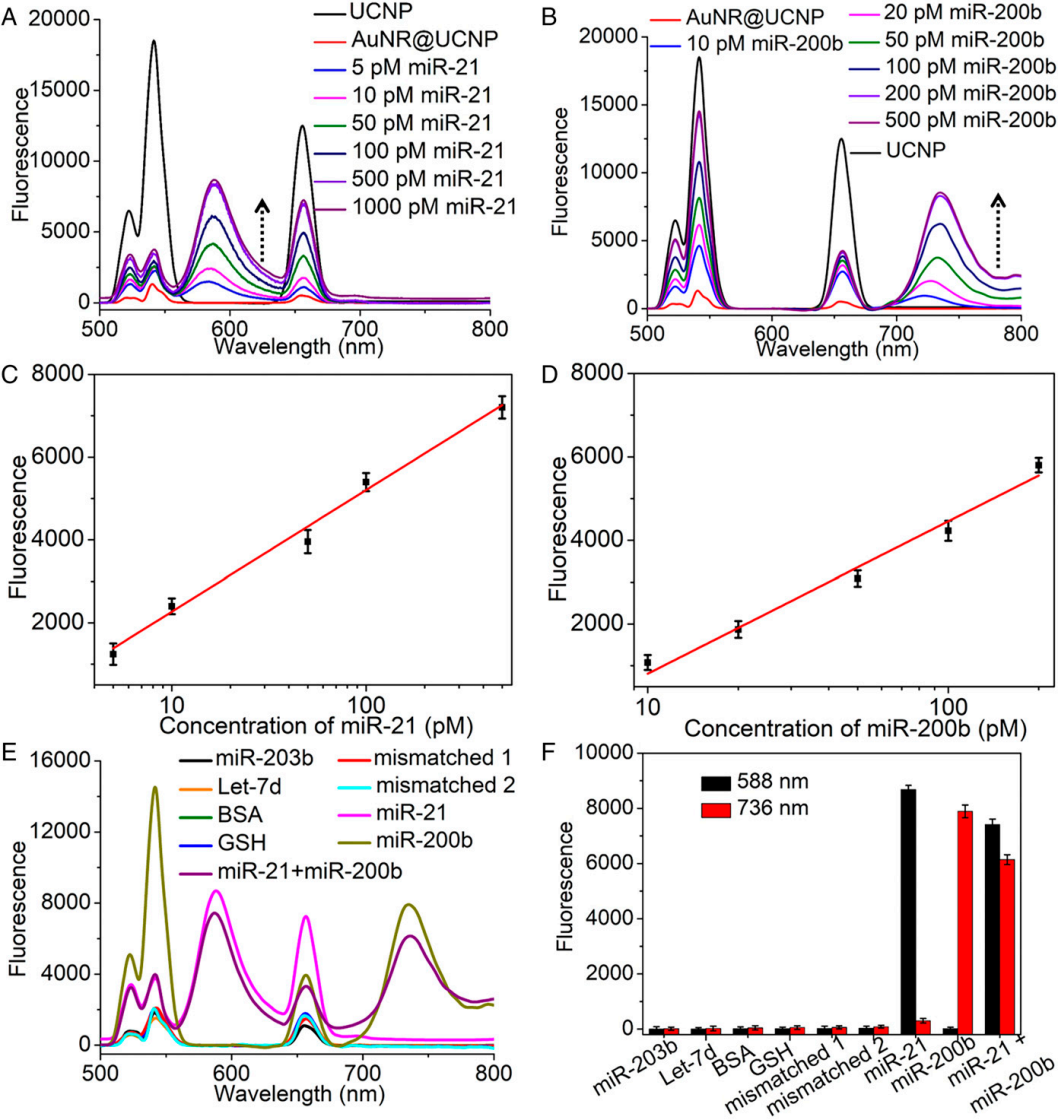
The AuNR@UCNP core–satellite assemblies used for dual miRNA detection in vitro. (*A*) Emission spectra of UCNP and AuNR@UCNP assemblies for different concentrations of miR-21 (0, 5, 10, 50, 100, 500, and 1,000 pM) in vitro (980-nm laser, 500 mW). (*B*) Emission spectra of UCNP and AuNR@UCNP assemblies for different concentrations of miR-200b (0, 10, 20, 50, 100, 200, and 500 pM) in vitro (980-nm laser, 500 mW). (*C*) Dependence of fluorescence intensity of TAMRA on different concentrations of miR-21 in vitro. (*D*) Dependence of fluorescence intensity of Cy5.5 on different concentrations of miR-200b in vitro. (*E*) Emission spectra and (*F*) emission intensity of AuNR@UCNP assemblies for miR-21, miR-200b, miR-21, and miR-200b, mismatched sequences of miR-21 (mismatched 1), mismatched sequences of miR-200b (mismatched 2), let-7d, miR-203^b^, BSA, and GSH in vitro (980-nm laser, 500 mW). Error bars in *C*, *D*, and *F* are mean ± SD (*n* = 3 independent samples).

To verify the feasibility of using the AuNR@UCNP for in vitro detection of miRNA, two pancreatic cancer markers, miR-21 and miR-200b, were used (53). When the dispersion of the NP assemblies was spiked with gradually increasing amounts of miR-21, TAMRA emission at 588 nm gradually increased, revealing a nearly perfect linear relationship vs. miR-21 concentration over a range of 5 to 1,000 pM (Fig. 3*A* and *SI Appendix*, Fig. S8). The emission at 540 nm increased slightly because the efficiency of LRET was limited. Also, the emission at 660 nm increased because the concentration of the dissociated UCNPs increased. When miR-200b was added, the emission intensity of Cy5.5 at 736 nm gradually increased (Fig. 3*B*), exhibiting a linear dependence with respect to the logarithmic concentration of miR-200b in the range of 10 to 500 pM (*SI Appendix*, Fig. S9). When the assembly solution was spiked simultaneously with miR-21 and miR-200b, the DNA hairpin captured the sequence by DNA–RNA hybridization and triggered the dissociation of the UCNPs from the AuNR cores, resulting in a detectable fluorescent signal and the intensities of peaks at both 588 and 736 nm simultaneously increased (*SI Appendix*, Fig. S10), retaining linear relationships with the concentrations of miR-21 and miR-200b (Fig. 3 *C* and *D*). These results confirm that the core–satellite assemblies of NPs responded sensitively to the two targets, with little cross-talk between them, which enables multiplexing in complex biological systems.

To demonstrate the analytical specificity of the AuNR@UCNP, the fluorescent responses induced by a mismatched sequence of miR-21 (mismatched 1, 100 pM), a mismatched sequence of miR-200b (mismatched 2, 100 pM), let-7d (100 pM), and miR-203^b^ (100 pM) were measured. No obvious signal changes were observed. In contrast, the addition of miR-21 (5 pM) or miR-200b (5 pM) led to a significant reduction in fluorescence intensity (Fig. 3 *E* and *F*). BSA and glutathione (GSH) were also included in the same experiments and the signal changes were negligible for both, suggesting that the detection method suffers little signal interference within a biological environment. Other nucleotides that have mismatching sequences have also been tested. No light emission was detected for sequences with different degrees of mismatch with the target RNA (*SI Appendix*, Fig. S11).

### Stability and Cytotoxicity of AuNR@UCNP Assemblies.

Although the core–satellite superstructures engineered with normal DNA can be lysed (*SI Appendix*, Fig. S12), self-assembled nanostructures from DNA strands attached to NPs via phosphorothioate bonds can resist enzymatic lysis in living cells (6–9, 44, 45). They are also stable in the presence of DNase I (0.1 U/μL, 37 °C for 2 h), indicating that AuNR@UCNP assemblies are lysis-resistant. The AuNR@UCNP assemblies displayed structural stability at the temperature of 20 °C and 40 °C in serum (*SI Appendix*, Fig. S13). UCL was quenched, and the fluorescence intensity of TAMRA and Cy5.5 at 559 and 685 nm had not been observed upon excitation at 980 nm (*SI Appendix*, Fig. S14), providing additional indication of the stability of the superstructure.

The effect of the AuNR@UCNP assembly on cell viability was also investigated. HeLa cells were incubated with different concentrations of AuNR@UCNP for 12 h. Cell viability decreased gradually when the assembly concentration exceeded 30 nM (based on the concentration of AuNRs; *SI Appendix*, Fig. S15). Therefore, AuNR@UCNP concentration of 30 nM when 92.8% of cells were viable was used in subsequent experiments. The results of a time-dependent cell viability test at an assembly concentration of 30 nM indicate negligible cytotoxicity throughout a period of 4 to 16 h (*SI Appendix*, Fig. S16). The cytotoxicity of PBS, AuNRs, UCNPs, and the AuNR@UCNP assembly were also investigated with Cell Counting Kit-8 (Beyotime), and no cytotoxicity was observed (*SI Appendix*, Fig. S17).

### Cellular Uptake of AuNR@UCNP Core–Satellite Assemblies.

Because mature miRNAs mainly exist in cytosol (1), it was important to facilitate the penetration of the AuNR@UCNP assemblies into the cells. For this purpose, the strands of TAT peptide were attached to the surface of the assemblies. The average number of TAT peptides on each AuNR@UCNP was about 630 ± 14. The detailed procedure for determination of the stoichiometry of TAT constructs with AuNR@UCNP is given in *SI Appendix*. Modified with TAT peptides, the assembly was still uniformly dispersed without aggregation (*SI Appendix*, Fig. S18). Moreover, the Zeta-potential of the assembly reveals a positive surface charge, which facilitates interactions with negatively charged cellular membranes to induce endocytosis (*SI Appendix*, Fig. S19). The bio-TEM images confirmed the successful internalization of AuNR@UCNP assemblies by HeLa cells. In accordance with a previous report (54), the NP constructs crossed the cellular membrane directly into the cytosol (*SI Appendix*, Fig. S20). Also, the confocal microscopy images of cells after incubation with AuNR@UCNP showed that the two dyes were present in their emissive state in the cytosol, indicating the analytical capabilities of AuNR@UCNP for the detection of intracellular miRNAs (*SI Appendix*, Fig. S21).

**Fig. 4.**
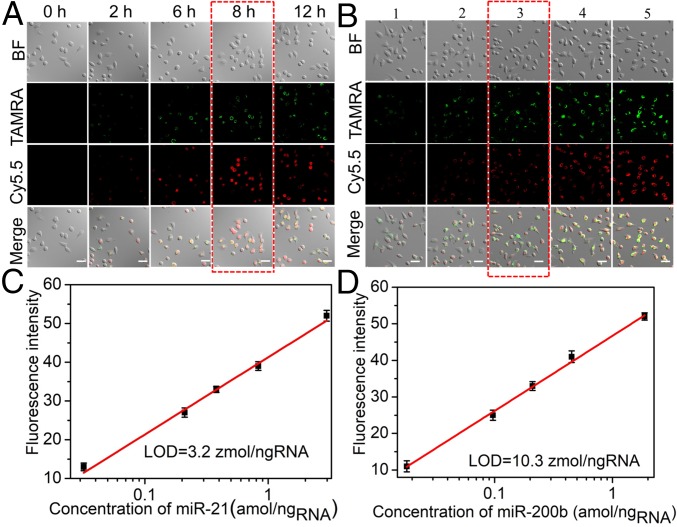
The AuNR@UCNP core–satellite assemblies for dual miRNA detection in living cells. (*A*) Confocal images of HeLa cells after incubation with AuNR@UCNP for different periods of time: 0, 2, 6, 8, and 12 h. (*B*) Confocal images of HeLa cells with different amounts of miR-21 and miR-200b: Columns 1 and 2 were transfected with antisense sequences of both miR-21 and miR-200b, column 3 was not transfected, and columns 4 and 5 were transfected with both miR-21 and miR-200b. (Scale bars: 20 µm.) (*C* and *D*) Emission intensity for different concentrations of intracellular miR-21 and miR-200b. A continuous-wave near-infrared laser operating at 980 nm was used for excitation; the power was 500 mW, and the images were collected at 588 ± 50 nm (green channel) and 736 ± 50 nm (red channel). Error bars in *C* and *D* are mean ± SD (*n* = 3 independent samples).

To investigate the uptake efficiency of the AuNR@UCNP assembly in living cells, HeLa cell cultures were incubated with 30 nM AuNR@UCNP for different periods of time (0 to 12 h). The cells were then washed three times with PBS to remove the culture medium and imaged by confocal microscopy acquiring the images for 588 ± 50 nm and 736 ± 50 nm under 980-nm laser excitation. The fluorescence intensities of TAMRA (green field) and Cy5.5 (red field) transferred from the UCNPs both peaked at 8 h in the living cells (Fig. 4*A*), and thus an 8-h incubation time was chosen as the detection time for intracellular miRNAs.

### Quantification of Two Types of miRNAs in Live Cells.

To determine the levels of miR-21 and miR-200b, HeLa cells were transfected with different amounts of miR-21 and miR-200b to increase their intracellular levels or with antisense miR-21 and miR-200b sequences (*SI Appendix*, Table S1) to reduce their levels, using a commercial transfection agent (Lipofectamine RNAiMAX Transfection Reagent). After treatment, intracellular miR-21 and miR-200b levels were measured with real-time RT-qPCR (*SI Appendix*, Figs. S22–S27). The transfected HeLa cells expressing different levels of miR-21 and miR-200b were incubated with AuNR@UCNP for 8 h. Confocal images of the TAMRA and Cy5.5 optical tags based on LRET reflected the expression levels of miR-21 and miR-200b, respectively. The emission intensities of both TAMRA and Cy5.5 increased as the amounts of intracellular miR-21 and miR-200b increased (Fig. 4*B*). Linearity between the emission intensity of TAMRA and the concentration of miR-21 spanned a range of 0.032 to 2.97 amol/ng_RNA_ (Fig. 4*C*), with the LOD calculated to be as low as 3.2 zmol/ng_RNA_ (0.11 amol or 6.5 × 10^4^ copies). To the best of our knowledge, this method represents the most sensitive detection protocol for intracellular miR-21 reported to date (*SI Appendix*, Table S2).

Similarly, a calibration curve for the emission intensity of Cy5.5 and the concentration of intracellular miR-200b covered the range of 0.018 to 1.87 amol/ng_RNA_; the LOD was 10.3 zmol/ng_RNA_ (0.34 amol or 2.1 × 10^5^ copies) (Fig. 4*D*). It is important to note that there was no previous method reported about miR-200b detection, monitoring, or imaging in living cells. Zeptomolar LODs are associated with the high yield of the AuNR@UCNP assemblies and the strong LRET efficiency between their constituent units. The emission of TAMRA and Cy5.5 is also very sensitive to the complementary miRNA segments, with no signal interference between them. To verify the collected signal at 588 ± 50 nm was not from UCNP, the confocal image acquired at 588 ± 20 nm is been displayed in *SI Appendix*, Fig. S28. The relative TAMRA fluorescence intensity values collected at 588 ± 50 nm and 588 ± 20 nm showed almost the same amplitude (*SI Appendix*, Fig. S29), indicating that the fluorescent signal at 588 ± 50 nm was the dye TAMRA. Furthering the concept of quantitative imaging of miRNA, fluorescence spectra of HeLa cells expressing different concentrations of miR-21 and miR-200b were also acquired. As shown in *SI Appendix*, Fig. S30, the fluorescence intensities at 588 nm and 736 nm both increased as the concentration of miR-21 and miR-200b increased. Importantly, the fluorescence intensity of TAMRA (588 nm) transferred from UCNP shows a linear relationship with amounts of intracellular miR-21 (*SI Appendix*, Fig. S31). The Cy5.5 fluorescence intensity (736 nm) concomitantly displayed a similarly linear relationship with amounts of intracellular miR-200b (*SI Appendix*, Fig. S32), enabling straightforward enumeration of concentrations of both miRNAs.

The results of experimental comparison between miR21-only AuNP@UCNP and miR200b-only AuNP@UCNP in living cells are shown in *SI Appendix*, Figs. S33 and S34. For HeLa cells transfected with different amounts of miR-21 only (miR21-only AuNP@UCNP), the fluorescence intensity of TAMRA (green field) increased with increased concentrations of miR-21, whereas the fluorescence intensity of Cy5.5 (red field) was weak and showed no changes because of the naturally occurring miR-200b in HeLa cells (*SI Appendix*, Fig. S33). The miR200b-only AuNP@UCNP displayed the same trend in living cells (*SI Appendix*, Fig. S34) as in the in vitro experimental series.

**Fig. 5.**
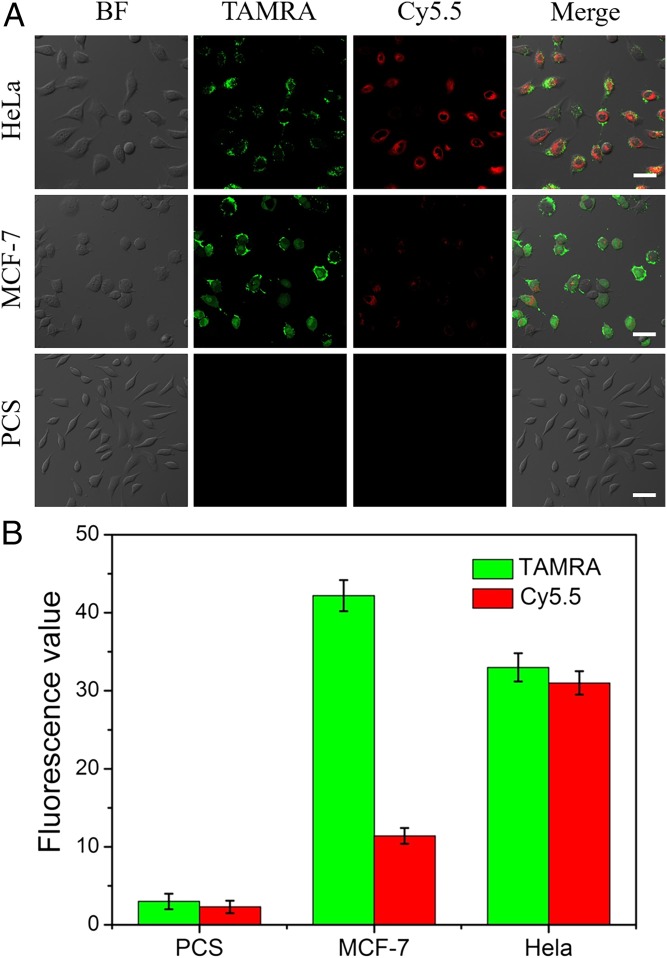
The AuNR@UCNP core–satellite assemblies used for dual miRNA detection in different cell lines. (*A*) Confocal images and (*B*) emission intensity values for PCS, MCF-7, and HeLa cells with AuNR@UCNP assemblies. A continuous-wave near-infrared laser operating at 980 nm was used for excitation; the power was 500 mW. (Scale bars: 20 μm.) Error bars in *B* are mean ± SD (*n* = 3 independent samples).

To test the practicality of AuNP@UCNP multiplexed miRNA detection, primary uterine fibroblast cells (PCS-460-010) and two cancer cell lines (HeLa and MCF-7) were used to simultaneously quantify the levels of miR-21 and miR-200b. Confocal images were taken when the AuNR@UCNP assemblies were added to the cells (Fig. 5*A*). The expression of miR-21 and miR-200b in normal PCS-460-010 cells was low; consequently, there was no signal observed from TAMRA or Cy5.5. The emission intensity of TAMRA was higher in the MCF-7 cells than in the HeLa cells, but the MCF-7 cells displayed a weaker fluorescence from Cy5.5 than the HeLa cells (Fig. 5*B*). According to the calibration curves, the amount of miR-21 detected was approximately three times higher in MCF-7 cells (1.12 amol/ng_RNA_) (38.5 amol) than in HeLa cells (0.38 amol/ng_RNA_) (13.1 amol) The calculated amounts of miR-200b were 0.17 amol/ng_RNA_ (5.6 amol) (MCF-7 cells) and 0.019 amol/ng_RNA_ (0.63 amol) (HeLa cells), respectively. These results are consistent with previously reported values (6–10) for both types of miRNA used here.

### Quantitative Imaging of Different Levels of miRNAs in Vivo.

**Fig. 6.**
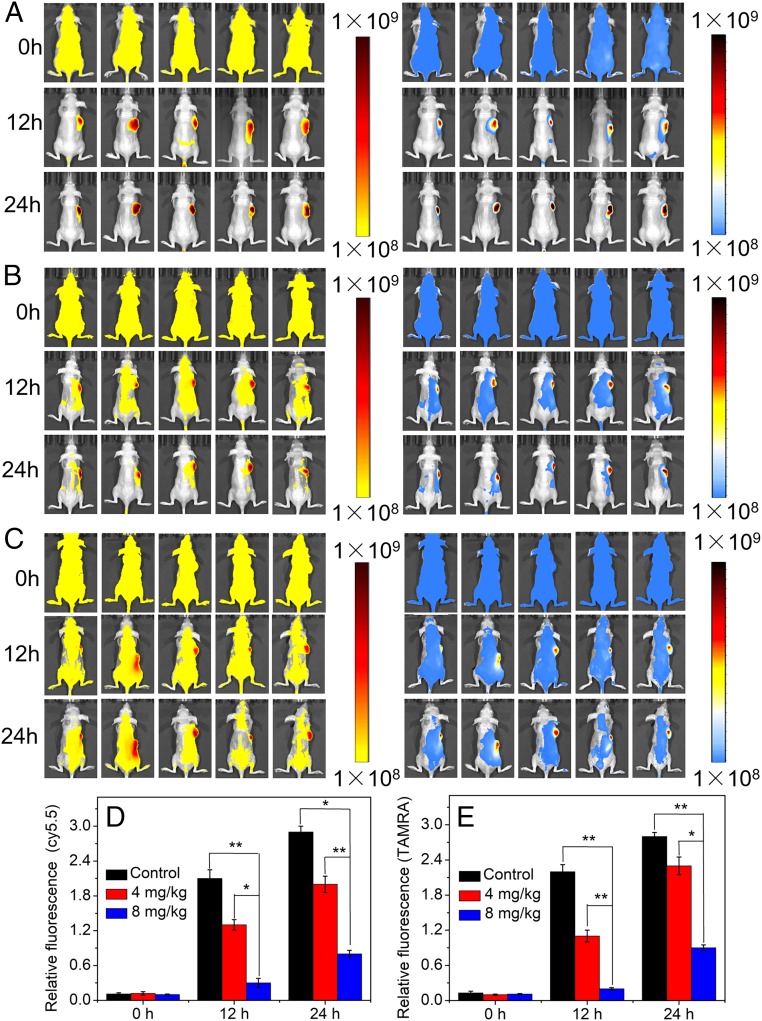
In vivo imaging of AuNR@UCNP core–satellite assemblies for dual miRNA detection. In vivo imaging of cancer with multiplexed miRNA markers. Ratiometric pseudocolor images of mice with different concentrations of miR-21 and miR-200b: (*A*) 0, (*B*) 4, and (*C*) 8 mg/kg miR-21 and miR-200b inhibitors were injected through the tail vein. Fluorescence images were acquired under 980-nm excitation. A continuous-wave near-infrared laser operating at 980 nm was used for excitation; the power was 500 mW; 736 ± 50 nm (blue channel) and 588 ± 50 nm (yellow channel) emission. (*D* and *E*) The relative Cy5.5 and TAMRA fluorescence intensity values of mice in tumor sites with different concentrations of miR-21 and miR-200b. Error bars in *D* and *E* are mean ± SD (*n* = 3 independent samples). *P < 0.05, **P < 0.01.

Once the feasibility of miRNA detection with AuNP@UCNP in living cell lines was confirmed, the same core–satellite assemblies were tested in mice bearing tumor xenografts. In a typical experiment, tumor xenografts were generated by the s.c. injection of female nude mice with HeLa cells. Upon tumor formation, the mice were randomly divided into three groups, with five mice per group. The mice were then treated with miRNA inhibitors to reduce the amounts of miR-21 and miR-200b at the tumor sites. Finally, the AuNR@UCNP assembly was injected into the mice through their tail veins and the fluorescent signals were measured at 736 ± 50 nm (blue channel) and 588 ± 50 nm (yellow channel), under laser excitation at 980 nm. Weak emission of Cy5.5 and TAMRA appeared in the tumor sections after 12 h and increased gradually after 24 h (Fig. 6), whereas negligible signals appeared in the other regions. Because of the overexpression of miR-21 and miR-200b in HeLa cells, the UCNPs dissociated from the assembly in the tumor region. Both the Cy5.5 fluorescent signal (blue channel) and the TAMRA fluorescent signal (yellow channel) were strong under 980-nm excitation (Fig. 6*A*). To analyze the levels of the miRNAs, different concentrations of miRNA inhibitors were injected into the mice, resulting in final doses of 4 mg miRNA inhibitor per kg body weight (Fig. 6*B*) and 8 mg miRNA inhibitor per kg body weight (Fig. 6*C*). The Cy5.5 and TAMRA fluorescent signals decreased as the amounts of injected miRNA inhibitors increased. MiR-21 and miR-200b are widely present in tumor sites and rarely found in other tissues and organs. Importantly, the NP assemblies could not be dissociated when accumulated in the liver, and thus no fluorescence was detected for this organ. The relative emission intensity of Cy5.5 were 2.0 and 0.8 when miR-200b–inhibitor doses were 4 mg/kg and 8 mg/kg, respectively (Fig. 6*D*), which were lower than those in the control group, treated with no miRNA inhibitor. The relative emission intensity values of TAMRA showed the same trends (Fig. 6*E*).

**Fig. 7.**
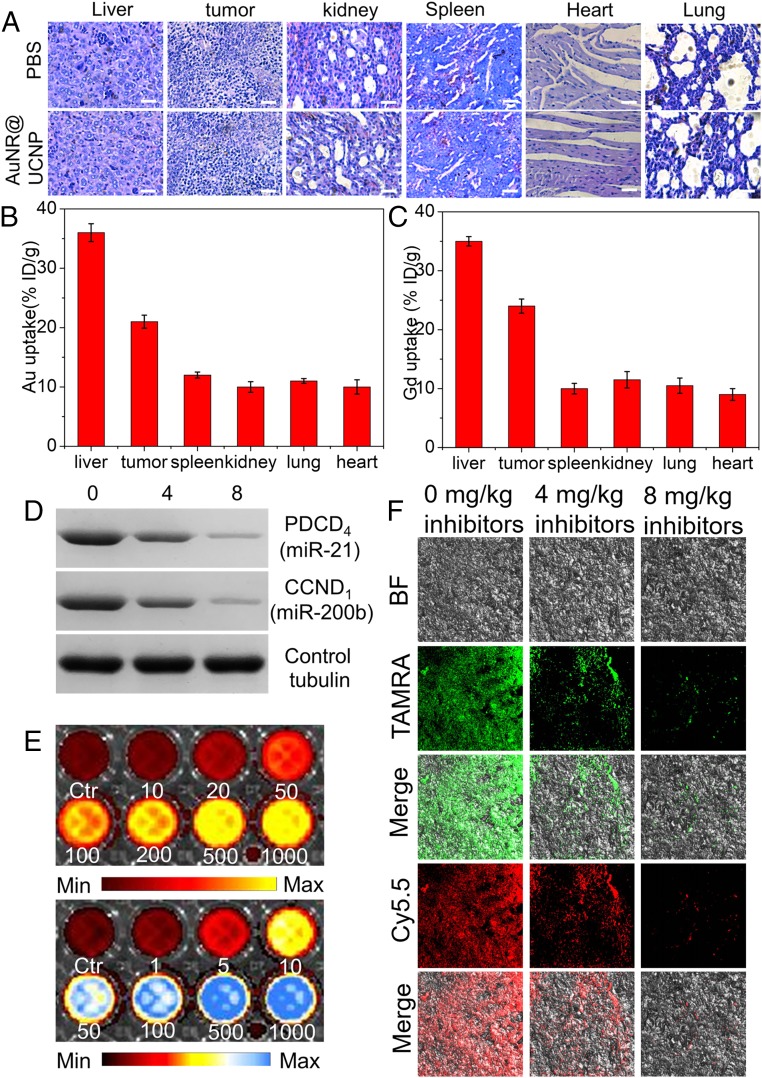
Dual miRNA detection in living mice with AuNR@UCNP assemblies. (*A*) Representative histological analysis of different organs after various treatments. (Scale bars: 100 μm.) Biodistribution of (*B*) Au and (*C*) Gd in tissue samples was measured by ICP-MS. (*D*) Western blot analysis of PDCD4, CCND1, and tubulin in mouse tumors in the presence and absence of miR-21 and miR-200b inhibitors. (*E*) Ratiometric pseudocolor images of AuNR@UCNP in 96-well plates treated with different concentrations (picomolar) of miR-21 and miR-200b using an in vivo imaging system, fluorescence images were acquired with for 980-nm excitation and 500-mW power, 736 ± 50 nm (blue channel) and 588 ± 50 nm (yellow channel) emission. (*F*) Histological analysis of tumor sections with different concentrations of miR-21 and miR-200b. Fluorescence images were acquired under 980-nm excitation and the power is 500 mW, 736 ± 50 nm (red channel) and 588 ± 50 nm (green channel) emission. Error bars in *B* and *C* are mean ± SD (*n* = 3 independent samples).

To evaluate the potential side effects of AuNR@UCNP assembly on the tissue, serum biochemistry tests were conducted. The alanine aminotransferase, aspartate aminotransferase, blood urea nitrogen, and creatinine analyses displayed levels of the corresponding biomarkers similar to those of the control group (*SI Appendix*, Fig. S35). No obvious liver damage or renal toxicity in the AuNR@UCNP groups can be therefore inferred. No significant damage was also detected in major organs (liver, kidney, heart, spleen, or lung) as indicated by the histological analysis (Fig. 7*A*).

The pharmacokinetics and biodistribution of AuNR@UCNP constructs were investigated in the mice by quantifying the Au and Gd content in major organs, tumors, and metabolites using inductively coupled plasma MS (ICP-MS). These elements accumulated mainly in the tumor and liver, indicating high biocompatibility and tumor-targeting capabilities of the AuNR@UCNP assemblies (Fig. 7 *B* and *C*). The half-life of AuNR@UCNP assemblies was calculated to be 1.3 h according the Au contents in blood (*SI Appendix*, Fig. S36). These data indicated that the AuNR@UCNP can be cleared quickly from the circulation. While Au and Gd exhibited greater accumulation in the liver and tumor than other organs (*SI Appendix*, Fig. S37 *A* and *B*), the amounts of Au and Gd elements in the major organs and tumors decreased greatly 5 d after injection. In 15 d, the levels of Au and Gd in the organ distribution were barely detectable. The changes in excretion of the same elements in feces and urine after tail vein injection were analyzed as well (*SI Appendix*, Fig. S37 *C* and *D*). The levels of Au and Gd in the feces and urine were gradually weakened and stabilized and mainly discharged through the feces 15 d after injection. Based on the above results, the AuNR@UCNP can be virtually completely cleared from the body in 15 d after injection.

To determine whether the miR-21 and miR-200b inhibitors reduced the target miRNAs, the relevant protein expression levels in tumor tissues were quantified with a Western blot analysis (55, 56). Tumor tissues treated with the highest concentrations of inhibitors showed the lowest expression of the PDCD_4_ and CCND_1_ proteins (Fig. 7*D*), which reflected the lowest levels of miR-21 and miR-200b, respectively. Ratiometric images of AuNR@UCNP, treated with different concentrations of miR-21 and miR-200b in a 96-well plate, were acquired with an in vivo imaging system, under 980-nm excitation. As the concentrations of miR-21 (0, 10, 20, 50, 100, 200, 500, and 1,000 pM) and miR-200b (0, 1, 5, 10, 50, 100, 500, and 1,000 pM) increased, both the TAMRA (yellow channel) and Cy5.5 (blue channel) fluorescence intensities were raised. The fluorescence intensities were the highest when the both concentrations reached 500 pM. Furthermore, no statistically significant changes in emission intensities were observed in the presence of higher concentrations of miR-21 and miR-200b (1,000 pM; Fig. 7*E*). These results indicated that greater number of UCNPs had dissociated from the assembly and more energy was transferred from UCL as the concentration of the targets increased. Note that the saturation detection concentrations were 500 pM. To decrease the amounts of miRNAs, different concentrations of miRNA inhibitors were injected into the mice. Then, the AuNR@UCNP (200 μL, 2 mg/mL, calculated in respect to the amount of AuNRs) was injected. After 24 h, the tumors were harvested and fixed in 10% formalin solution. A histological analysis of tumor sections from mice treated with different concentrations of miR-21 and miR-200b confirmed these results (Fig. 7*F*). The lowest emission intensities were observed for the largest amounts of miRNA inhibitors injected. Therefore, this high targeting ability and detection sensitivity could be used for diagnosis and treatment of early-stage tumors (57–59).

## Conclusions

The simultaneous quantification of two miRNAs is realistic for both in vitro and in vivo analyses using engineered NP–nanorod assemblies with generalized structure of AuNP@UCNP. The LOD values for miR-21 and miR-200b in living cells were as low as 3.2 zmol/ng_RNA_ (0.11 amol or 6.5 × 10^4^ copies) and 10.3 zmol/ng_RNA_ (0.34 amol or 2.1 × 10^5^ copies), respectively, which enabled miRNA detection and quantification of miRNA cancer markers in mice. These findings and the versatile construction of NP assemblies engenders broad opportunities in utilization of miRNA for fundamental epigenetics and clinical diagnosis.

## Materials and Methods

*SI Appendix* describes all experimental procedures, such as synthesis of Au NRs and UCNPs, assembly of AuNRs and UCNPs core–satellite superstructures, details on miR-200b and miR-21 detection in vitro and in vivo, and histopathological examination. More details are in *SI Appendix*, Figs. S1–S37. DNA and RNA sequences used in NP assembly and miRs detection are also available (*SI Appendix*, Tables S1 and S2).

All animal studies were performed according to institutional ethical guidelines and were approved by the Committee on Animal Welfare of Jiangnan University.

## Supplementary Material

Supplementary File
