# Supplementary material for: Quantitative zeptomolar imaging of miRNA cancer markers with nanoparticle assemblies
Source: Proc Natl Acad Sci U S A. 2019 Feb 11;116(9):3391–400. doi: 10.1073/pnas.1810764116 (PMC6397542; doi:10.1073/pnas.1810764116)
Supplement: Supplementary File [file pnas.1810764116.sapp.pdf]

www.pnas.org

Supplementary Information for

Quantitative Zeptomolar Imaging of Micro RNA Cancer Markers with Nanoparticle Assemblies

Aihua Qu[ab1], Maozhong Sun[ab1], Liguang Xu[ab], Changlong Hao[ab], Xiaoling Wu[ab], Chuanlai Xu[ab], Nicholas A. Kotov[cd2], Hua Kuang[ab2]

[a] State Key Lab of Food Science and Technology, School of Food Science and Technology, Jiangnan University, 214122, Wuxi, Jiangsu, China; [b] International Joint Research Laboratory for Biointerface and Biodetection, Jiangnan University, 214122, Jiangsu, China; [c] Department of Chemical Engineering, Biointerface Institute, University of Michigan, Ann Arbor, MI, 48109, USA; [d] Michigan Institute of Translational Nanotechnology, Ypsilanti, MI, 48198.

The authors declare no competing financial interest.

[1]A.Q. and M.S. contributed equally to this work.

[2]To whom correspondence should be addressed. Email: kuangh@jiangnan.edu.cn; kotov@umich.edu;

This PDF file includes:

Supplementary text
Figures. S1 to S37
Tables S1 to S2
References for SI reference citations

www.pnas.org/cgi/doi/10.1073/pnas.1810764116

Supplementary Information Text

Material and instrument. The thiolated DNA oligonucleotides, purified with high-performance liquid chromatography (purity > 95%), were manufactured by Shanghai Sangon Biological Engineering Technology & Services Co. Ltd, and then suspended in TE buffer (Shanghai Sangon) at a final concentration of 100 μM, miR-21, antisense miR-21, miR-200b, antisense miR-200b, and two mismatched oligonucleotides that were purchased from Shanghai GenePharma Co. Ltd (Shanghai, P.R. China). All the reagents used in this study were purchased from Sigma-Aldrich (St. Louis, MO, USA). The deionized (DI) water used throughout the study was obtained with a Milli-Q device (18.2 MΩ; Millipore, Molsheim, France). All glassware was cleaned with freshly prepared aqua regia and rinsed thoroughly with DI H_2O before use. The HeLa, MCF-7, and PCS cell lines were obtained from the China Center for Type Culture Collection (Wuhan, P.R. China).

Transmission electron microscopy (TEM) images were obtained with a JEOL JEM-2100 microscope at an acceleration voltage of 200 kV. The particle size distributions were measured with a Zetasizer Nano ZS system (Malvern) with a 633 nm laser. The ultraviolet–visible (UV–Vis) spectra were obtained with a UNICO 2100 PC UV–Vis spectrophotometer and processed with OriginLab software. UCL spectra were obtained with a Hitachi F-7000 fluorescent spectrometer with an external 980 nm laser as the excitation source. Confocal images of cells were acquired with a modified Olympus FV1000 laser scanning upconversion luminescence microscope equipped with a continuous-wave laser at 980 nm (Connet Fiber Optics, China). A 60 × oil-immersion objective lens was used. The laser provided excitation at 980 nm, and the emitted luminescence was collected at 736 ± 50 nm and 588 ± 50 nm. In vivo images of tumors were obtained with the Maestro™ In-Vivo Fluorescent Imaging System (CRi Inc.) using a 980-nm-optical-fiber-coupled laser as the excitation source.

Method

Synthesis of gold nanorods. Gold nanorods were synthesized using a seed-mediated growth method. The seed solution was prepared as follows: 5 mL of 0.5 mM $HAuCl_4$ and 5 mL of 0.2 M CTAB solution were mixed together in a 20 mL scintillation vial. Then 0.6 mL of fresh 0.01 M $NaBH_4$ was added to the mixture with vigorous stirring (1200 rpm). Stirring was stopped after 2 min, and the solution color changed from yellow to brownish yellow. The solution was incubated at room temperature for 30 min before use.

To prepare the growth solution, 0.617 g of sodium oleate and 3.5 g of CTAB were dissolved in 125 mL of warm water. When the solution had cooled to 30 °C, 125 mL of 1 mM $HAuCl_4$ solution and 9 mL of 4 mM $AgNO_3$ solution were added. The solution was stirred at 700 rpm for 90 min, and 0.75 mL of HCl (37 wt% in water, 12.1 M) was added to adjust the pH. After another 15 min of stirring (400 rpm), 0.625 mL of 0.064 M ascorbic acid was introduced with vigorous stirring for 30 s. Finally, 0.2 mL of the seed solution was added to the growth solution. The resultant mixture was stirred for 30 s and left undisturbed at 30 °C for 12 h to allow nanorod growth.

Preparation of $NaGdF_4$: Yb and Er upconverting nanoparticles (UCNPs). The UCNPs (20 ± 3 nm) were synthesized according to the previously reported method, with slight

modifications. (1) $GdCl_3 \cdot 6H_2O$ (0.80 mmol), $YbCl_3 \cdot 6H_2O$ (0.18 mmol), and $ErCl_3 \cdot 6H_2O$ (0.02 mmol) were dissolved in a mixture of oleic acid (OA) (14 mL) and octadecene (ODE) (16 mL) in a flask. The solution was heated to 150 °C under nitrogen protection to form a homogeneous solution. Then, methanol solution (10 mL) containing NaOH (0.100 g, 2.5 mmol) and NH_4F (0.148 g, 4 mmol) were added slowly. After it had cooled to room temperature, the reaction mixture was stirred at 100 °C under vacuum for 10 min to remove the methanol, and then heated to 320 °C under nitrogen protection and maintained for 1 h. The reaction was terminated by cooling the reaction mixture to room temperature. The resultant nanoparticles, $NaGdF_4$, were precipitated with ethanol, collected by centrifugation, washed several times with ethanol, and then redispersed in tetrahydrofuran (THF) for subsequent experiments.

PEG-phosphate ligand (100 mg) was mixed with the OA-coated $NaGdF_4$ nanoparticles (10 mg) in THF (10 mL). The reaction mixture was maintained at room temperature overnight with stirring. The PEG-coated particles were washed three times with cyclohexane, and then dried under vacuum at room temperature. The PEG-coated $NaGdF_4$ particles obtained in this way dissolved readily in aqueous media, independently of the particle size.

DNA functionalization of AuNRs and UCNPs. The prepared AuNRs were concentrated 10-fold in 0.005 M CTAB, and then modified with DNA_2 (10 μM, dissolved in TE buffer) in a DNA:AuNR molar ratio of 10:1. The mixture was allowed to stand at room temperature for 12 h. The modified AuNRs were placed in a centrifuge and spun at 6000 rpm for 5 min (three times) to remove any excess DNA, and then resuspended in 0.001 M CTAB. DNA_5 was then added to the AuNRs sample in a 100:1 molar ratio, and the solution was incubated for 12 h with shaking at room temperature. The conjugates were purified by centrifugation at 6000 rpm for 5 min to remove the nonconjugated DNA, and resuspended in phosphate buffer (10 mM, pH 7.4) containing 10% CTAB.

The water-soluble UCNPs (10 mM) were diluted to 10 nM in phosphate buffer (10 mM, pH 7.4). One half of the UCNPs were functionalized with DNA_1 in a molar ratio of 5:1. The mixture was incubated for 12 h, and ultrafiltered (30 kDa molecular-weight cut-off) to remove the nonconjugated DNA. TAMRA modified-DNA3 was then added to the mixture to hybridize with DNA_1. After incubation for 12 h, the final UCNP bioconjugate was purified using ultracentrifugation at 8000 g for 10 min, suspended in PB buffer and stored at 4 °C. The other half of the UCNPs were functionalized with DNA_4 and DNA_6 under the same conditions.

Assembly of AuNRs and UCNPs into core-satellite superstructures. The purified DNA_2, DNA_5-modified AuNR core, the DNA_1, and DNA_3-modified UCNPs or DNA_4, and DNA_6-modified UCNPs satellites in a molar ratio of 1:20:100 were mixed in hybridization buffer (10 mM PBS [pH 7.4], 5 mM $MgCl_2$, 0.01% Tween 20). The mixture was incubated in a 60 °C water bath for 60 min and then cooled to 37 °C for 12 h. When the assembly of the AuNR@UCNP core-satellite was complete, thiolated PEG (5 kDa) was added to the core-satellite solution in a molar (PEG:AuNR core) ratio of 3000:1 and kept at 60 °C for 30 min in a water bath. The modified core-satellite assembly was then placed in a centrifuge at 6000 rpm for 5 min, and then suspended in PBS.

miR-200b and miR-21 detection in vitro. To determine the detection sensitivity of the assembly in vitro, the assembly in 10 mM PBS buffer was spiked simultaneously with different concentrations of miR-200b and miR-21. After incubation for 1 h at 37 °C, the fluorescent signals were obtained under 980 nm excitation and the power is 500 mW.

To measure the feasibility and selectivity of the AuNR@UCNP core-satellite assembly, the target miRNAs (miR-200b and miR-21), miR-203[b], let-7d, bovine serum albumin (BSA), glutathione (GSH), and mismatched sequences were added to the detection solution for 1 h (24 h for the BSA and GSH samples) at 37 °C, and the fluorescence was measured after the reaction. Fluorescence signals were acquired under 980 nm excitation and the power is 500 mW.

Cell lines and incubation conditions. HeLa (human epithelial carcinoma) cells, MCF-7 (human breast cancer) cells, and PCS-460-010 (normal human epithelial) cells were purchased from the American Type Culture Collection (Manassas, VA). The cells were cultured in Dulbecco's modified Eagle's medium containing 10% fetal bovine serum and 1% penicillin/streptomycin at 37 °C under a 5% CO_2 atmosphere. The cell numbers were counted with a Petroff–Hausser cell counter (USA). Serum-free medium (Opti-MEM; Life Technologies) was used for the transfection process.

Cell viability assessment. To evaluate the cytotoxicity of the AuNR@UCNP assembly, HeLa cells were seeded in RPMI-1640 medium with 10% fetal bovine serum in a 96-well plate, with five wells for each concentration of the assembly. The cells were incubated at 37 °C for 24 h in culture medium. Different concentrations of the assembly, modified with cell penetrating peptides (TAT), were then added to the culture medium and incubated for 24 h. The culture medium was replaced with 100 μL of fresh Opti-MEM (Life Technologies) and the cells were treated with 10 μL of Cell Counting Kit-8 (CCK-8, Beyotime) for 4 h. The absorbance (A) of each well was measured on a microplate reader at 450 nm, and the relative cell viability (%) was calculated as $(A_{test}/A_{control}) \times 100$. The viability of cells treated with the assembly for different incubation periods was also measured with the CCK-8 assay.

HeLa cells were seeded in a 35 mm Petri dish at a density of 10^4 cells per well for 24 h. The cells were incubated with 200 μL of various assemblies for 24 h at 37 °C. The excess assemblies were removed and the cells were washed with PBS, and then fixed with 4% paraformaldehyde in PBS for 15 min. The cells were co-stained with the LIVE/DEAD Cell Imaging Kit (Thermo Fisher Scientific Inc.) for 30 min, washed with PBS, and examined with Leica laser scanning confocal microscopy with 488 nm and 552 nm laser irradiation, and the power is 200 mW.

Quantitation of number of TAT peptides per AuNR@UCNP. The number of TAT peptides that are conjugated to the AuNR@UCNP can be measured using the following procedure.(2, 3)

(1) Label TAT peptide with FAM fluorescent dyes using a standard labeling kit.

(2) Then, determine a linear calibration curve that relates the concentration of a fluorescently labeled TAT peptide and fluorescence intensity.

(3) TAT peptide solution (5 mM) was added, with a peptide- to- AuNR@UCNP ratio of 1000:1. The solution was incubated for 24 h followed by removal of the un-coupled peptide though centrifugation. The fluorescence signals from the supernatant at the 490 nm excitation/518 nm emission wavelengths of the FAM were used to tag TAT peptide.

(4) Use the calibration curves to determine the concentration of TAT peptide in the supernatant. Subtract these values from the initial concentrations to obtain the amount of TAT peptide that is attached to AuNR@UCNP.

(5) To measure the initial concentrations, prepare TAT peptide solutions at exactly the same concentrations as in the reaction mixture with AuNR@UCNP.

Calculating intracellular miR-21 and miR-200b in Hela cells. The synthetic miR-21 and miR-200b were measured with the TaqMan® MicroRNA Assay (Fig. S15, Fig. S17), and standard curves of the cycle threshold values (Ct) versus the concentrations of miR-21 and miR-200b were constructed (Fig. S16, Fig. S18). The RT-qPCR amplification plots of the miR-21 and miR-200b extracted from the regulated cells were constructed, and the C_t values for miR-21 and miR-200b were then read from Fig. S19 and Fig. S20, respectively. The amounts of miR-21 and miR-200b in the six samples were calculated from the standard curves in Fig. S16 and Fig. S18, respectively.

Transfection of Hela cells with miR-200b and miR-21 and their antisense sequences. HeLa cells, cultured for 12 h in culture plates with (initially) 1.0×10^6 cells, were transfected with different amounts of synthetic miR-21 and miR-200b, or antisense miR-21 and miR-200b. The transfection experiment was performed with Lipofectamine® RNAiMAX Transfection Reagent (Life Technologies), according to manufacturer's instructions. Every cell medium was replaced with 10 mL of Opti-MEM containing RNAiMAX (300 μL) and the cells were transfected with miR-21 (1, 10 pM) and miR-200b (2, 10 pM), or antisense miR-21 (1, 10 pM) and miR-200b (2, 10 pM) for 24 h. After transfection, the intracellular miR-21 and miR-200b concentrations were quantified using a commercially available miR-specific qRT–PCR kit, and then for the following experiments.

Quantification of miR-200b and miR-21 in transfected HeLa cells. The miR-200b and miR-21 in the transfected cells were extracted with the PureLink® RNA Mini Kit (Life Technologies) and quantified with the TaqMan® Small RNA Assay and ABI Prism® 7900HT PCR (Life Technologies).

Confocal imaging of cellular miR-200b and miR-21. The transfected HeLa cells expressing different levels of miR-21 and miR-200b were cultured on glass-bottomed Petri dishes (for confocal microscopy; Nest Biotechnology Co., Ltd) for 12 h, at an initial density of 2×10^4 cells/dish. Then, the cells were incubated with the AuNR@UCNP probe for 8 h, After culture, the cells were washed three times with cold PBS to remove any extracellular assembly. Confocal images of the treated cells were obtained with confocal fluorescence microscopy. The fluorescence of the assembly was excited at 980 nm and the power is 500 mW, the fluorescent images were collected at 588 ± 50 nm and 736 ± 50 nm. The experiments were repeated three times with similar results.

Animal tumor models. All animal experiments conformed to the guidelines of the Chinese Animal Use and Care Committee. Five-week-old female nude mice were subcutaneously inoculated above the right flanks with 5×10^6 HeLa cells suspended in 50 μL of cold PBS. The in vivo studies were performed after 3 weeks.

Delivery of miR-200b and miR-21 inhibitors in vivo. The mirVana™ miRNA Inhibitors were complexed with Invivofectamine 3.0 Reagent (Life Technologies) for in vivo applications. The miRNA oligonucleotides (2 mg/mL or 3.5 mg/mL in 750 μL of water) were mixed with the manufacturer's complexation buffer (750 μL), and then 1500 μL of Invivofectamine 3.0 Reagent was added. After incubation for 30 min at 50 °C, dialysis was performed against 4 L of PBS to remove excess salts and solvents. The resulting miRNA inhibitor concentration was 0.4 or 0.8 mg/mL. A 200 μL injection into the tail vein of each 20 g mouse, resulted in a final miRNA dose of 4 or 8 mg/kg body weight.

In vivo imaging of miRNAs. UCNP luminescent imaging was performed with an IVIS Lumina II in vivo imaging system (Caliper Life Sciences, Inc.) at various time points (0, 12, or 24 h). The AuNR@UCNP assembly (200 μL, in terms of the amount of AuNR, 2 mg/mL) was intravenously injected into the nude mice. After 0 h, 12 h, and 24 h, fluorescent images of the flanks of the live mice were taken under a 980 nm laser excitation and the power is 500 mW.

Western Blot analysis. In the Western Blot analysis, tumor tissues were lysed with RIPA Lysis Buffer (Beyotime). The protein lysates were separated with SDS-PAGE and then transferred to polyvinylidene difluoride (PVDF) membranes (Life Technologies). The membranes were incubated with primary antibody against $PDCD_4$ or $CCND_1$ (diluted 1:1000), and then with a horseradish-peroxidase-conjugated secondary antibody (1:1000 dilution). Tubulin (1:1000 dilution) was used as the loading control.

Histopathological examination. After the delivery of different concentrations of miRNA inhibitors, the AuNR@UCNP assembly (200 μL, 2 mg/mL, in terms of the amount of AuNR) was injected through the tail veins of the mice. After 24 h, the tumors were harvested and fixed in 10% formalin solution for 12 h. The prepared tumor samples were refrigerated, sliced into 5 μm sections, and mounted on glass slides. Images of the treated tumor sections were obtained with confocal fluorescence microscopy. Fluorescence images were acquired with for 980 nm excitation and 500 mW power.

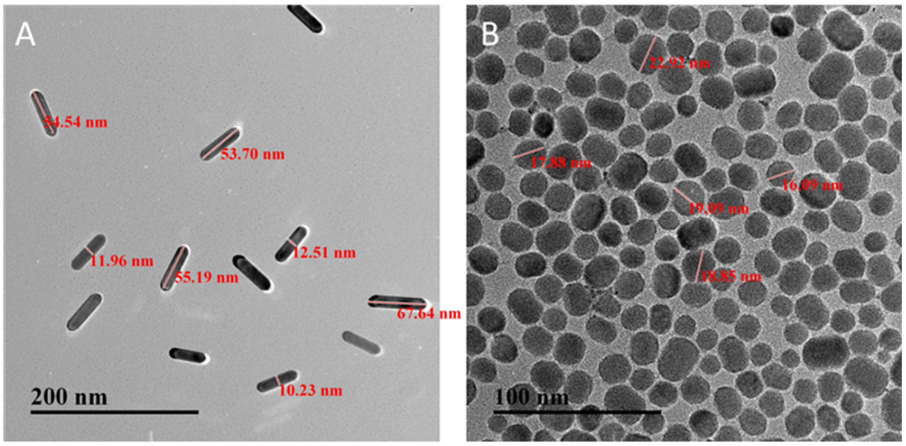

Fig. S1. Representative TEM images of (A) AuNRs and (B) UCNPs.

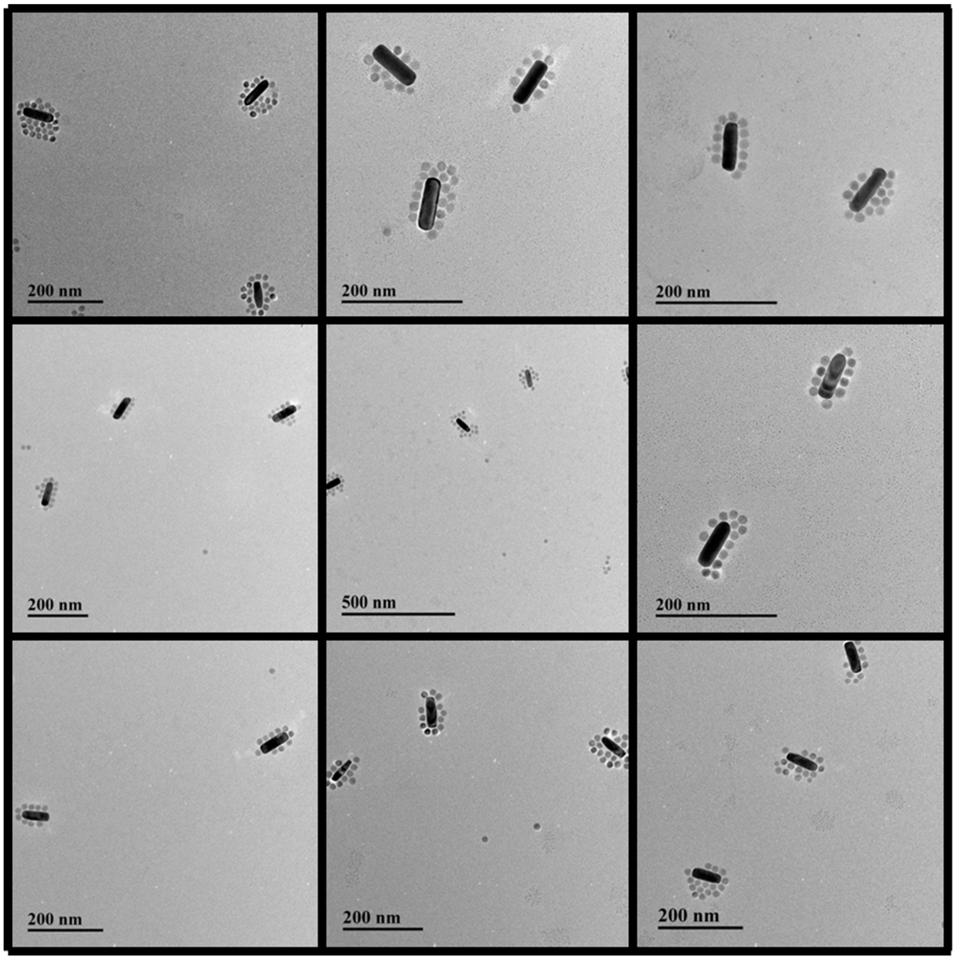

Fig. S2. TEM images of AuNR@UCNP assemblies in buffer.

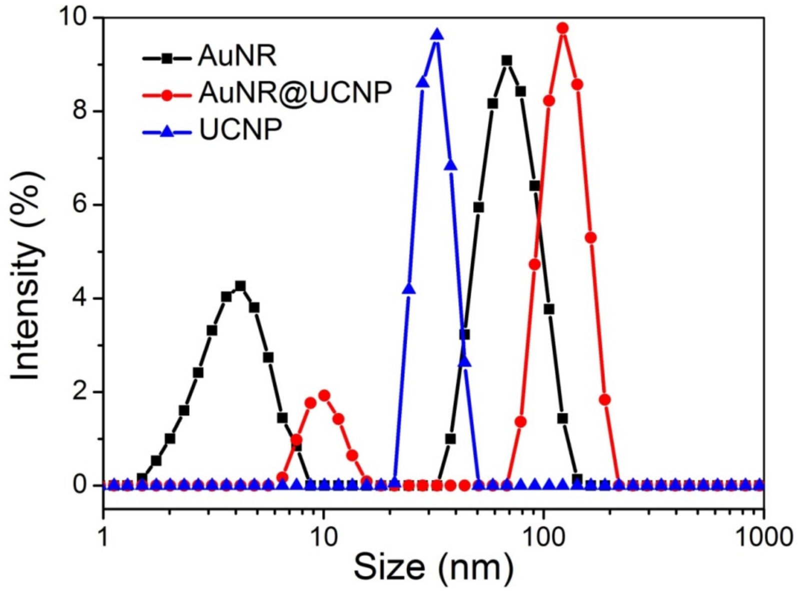

Fig. S3. DLS intensity of AuNRs, AuNR@UCNP and UCNPs.

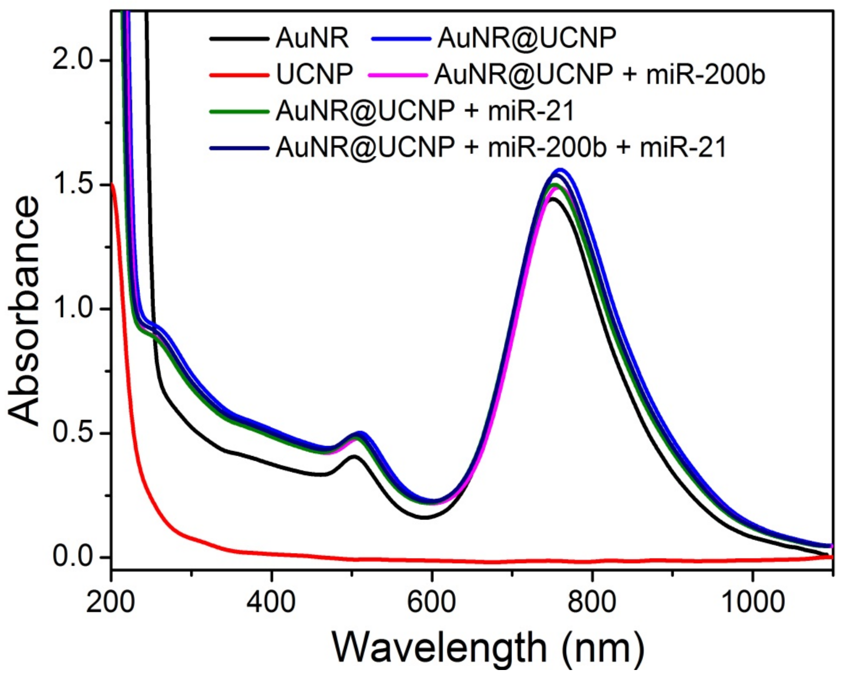

Fig. S4. UV-Vis absorption spectra of AuNR, UCNP, AuNR@UCNP, AuNR@UCNP with miR-21, AuNR@UCNP with miR-200b, and AuNR@UCNP with miR-21, miR-200b.

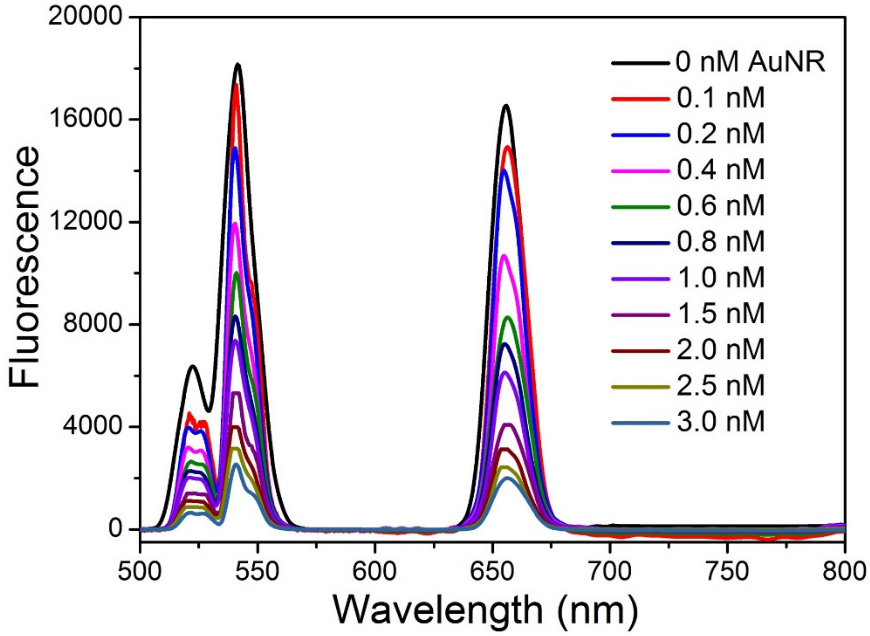

Fig. S5. UCL spectra of the assembly at varying concentration ratios of AuNR over UCNPs in PBS buffer, the concentration of UCNPs was 0.1 mg/mL (980nm laser, 500 mW).

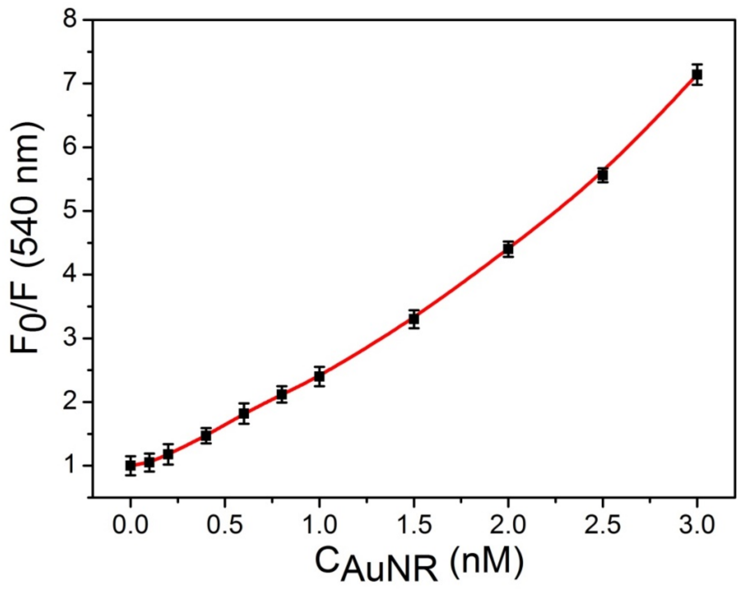

Fig. S6. The quenching degrees of AuNR over UCNPs at 540nm in PBS buffer. The data are presented as the mean ± s.d. (n = 3).

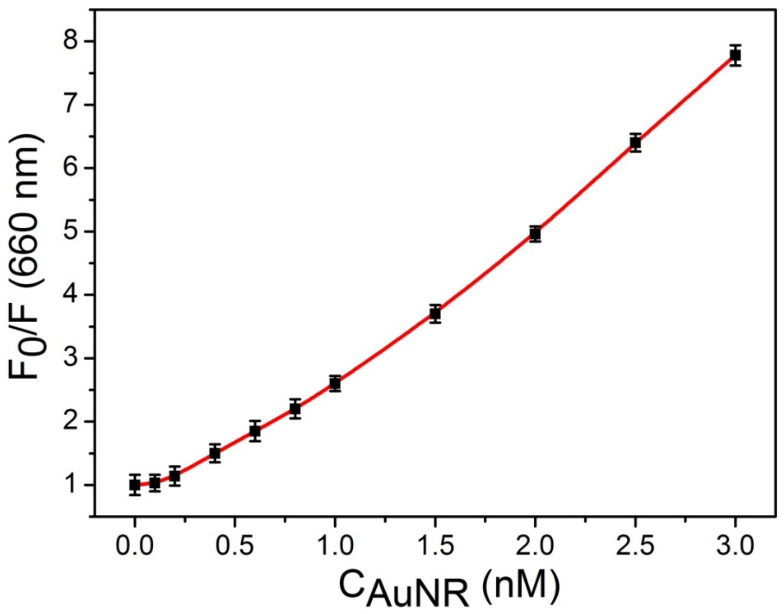

Fig. S7. The quenching degrees of AuNR over UCNPs at 660nm in PBS buffer. The data are presented as the mean ± s.d. (n = 3).

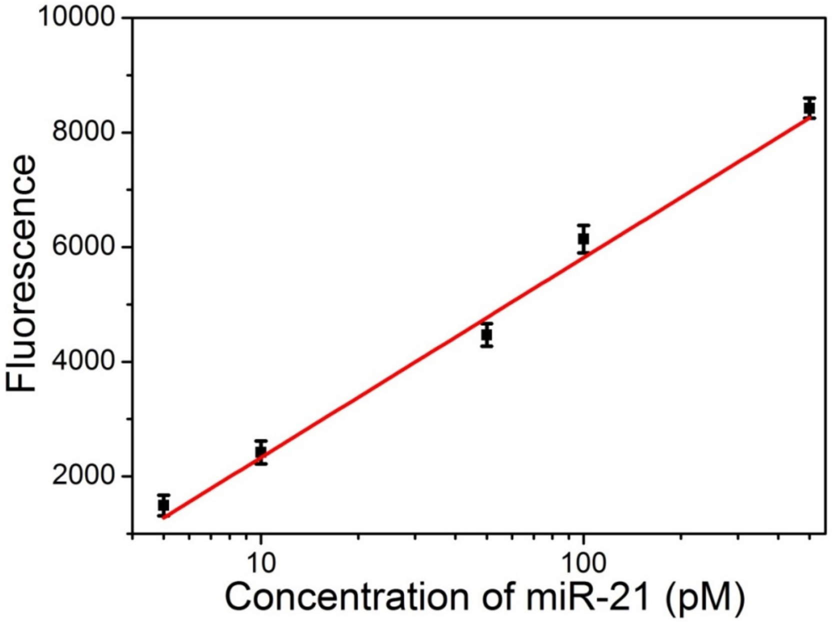

Fig. S8. Linear correlation of fluorescence intensity (TAMRA) versus different concentrations of miR-21 in vitro. The data are presented as the mean ± s.d. (n = 3).

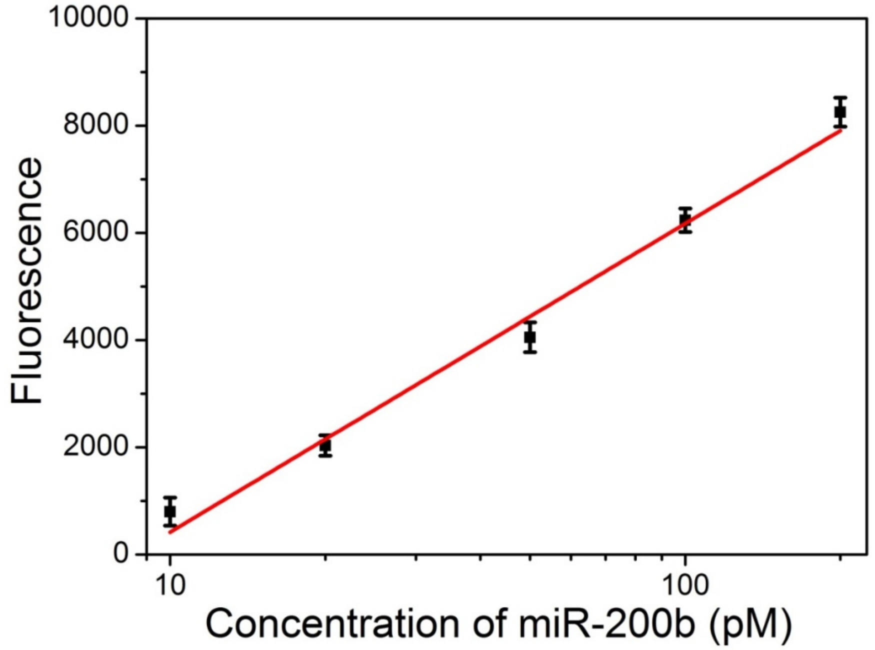

Fig. S9. Linear correlation of fluorescence intensity (Cy5.5) versus different concentrations of miR-200b in vitro. The data are presented as the mean ± s.d. (n = 3).

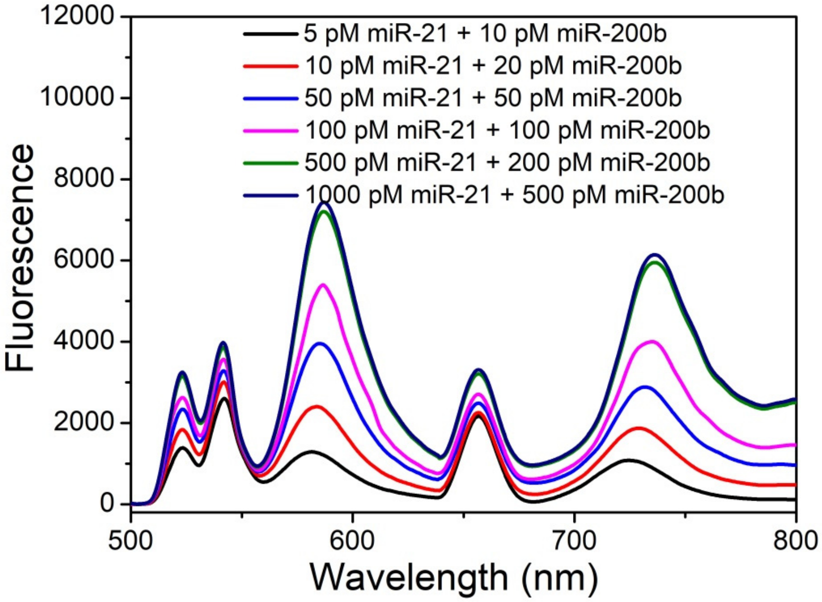

Fig. S10. Fluorescence spectra of AuNR@UCNP assemblies as responded to different concentrations of miR-21 (5, 10, 50, 100, 500, and 1000 pM) and miR-200b (10, 20, 50, 100, 200, and 500 pM) in vitro (980nm laser, 500 mW).

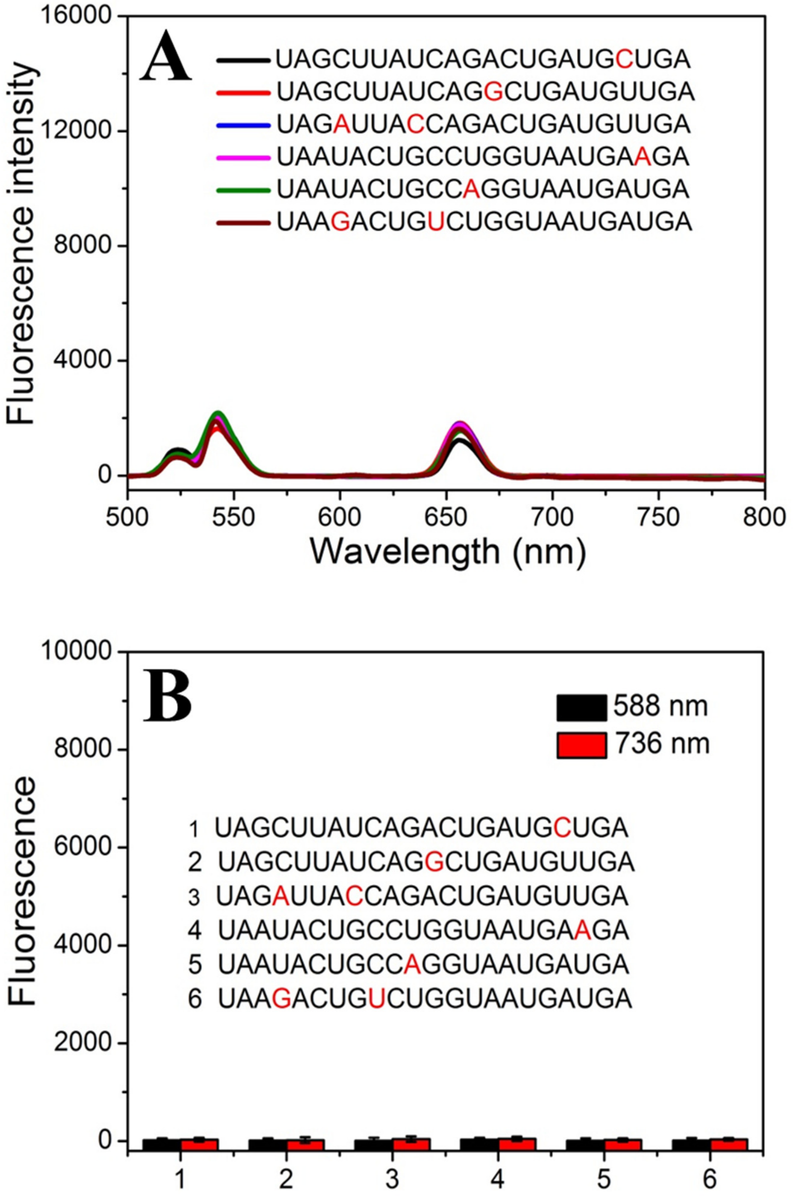

Fig. S11. (A) Fluorescence spectra of AuNR@UCNP assemblies as responded to other mismatched sequences. (B) Fluorescence intensity of AuNR@UCNP assemblies as responded to other mismatched sequences (980nm laser, 500 mW). The data are presented as the mean ± s.d. (n = 3).

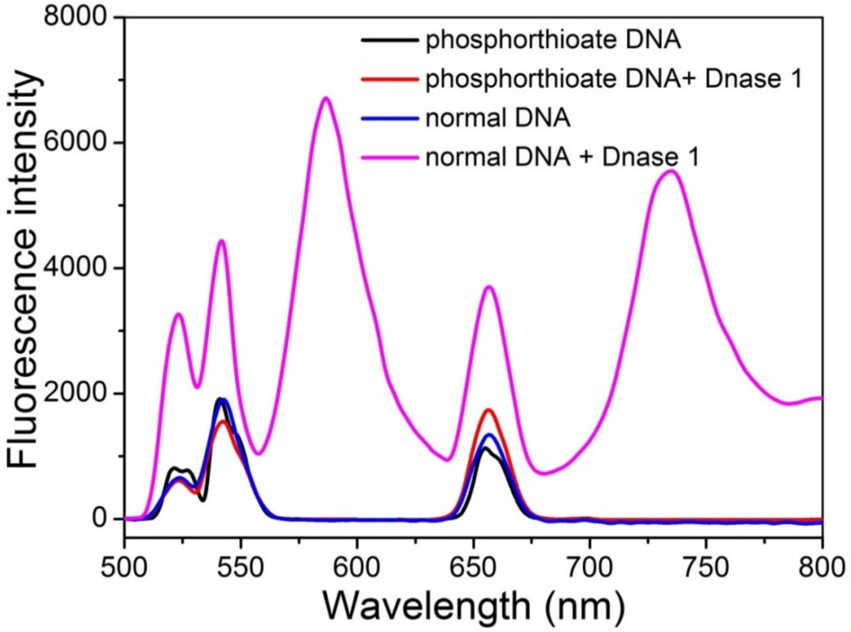

Fig. S12. Stability of AuNR and UCNP core-satellite constructed with phosphorthioate DNA. Fluorescence spectra is acquired under 980 nm excitation and the power is 500 mW.

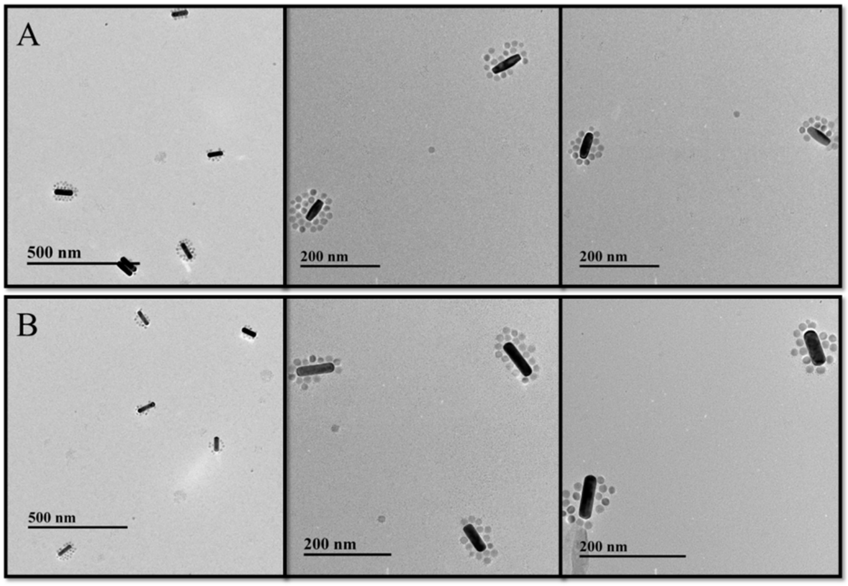

Fig. S13. TEM images of the AuNR@UCNP assembly in the serum with different temperature, A: 20 °C and B: 40 °C.

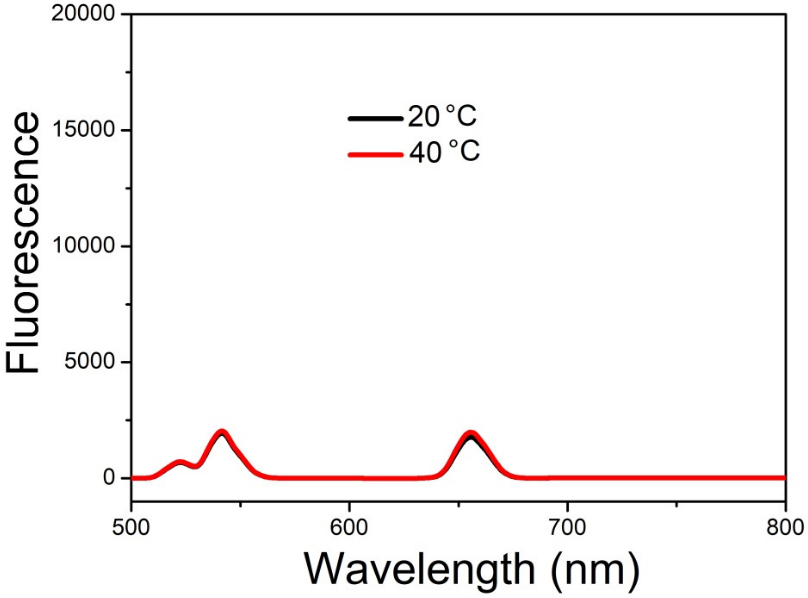

Fig. S14. Fluorescence spectra of the AuNR@UCNP assembly in the serum with different temperature under 980 nm excitation, and the power is 500 mW.

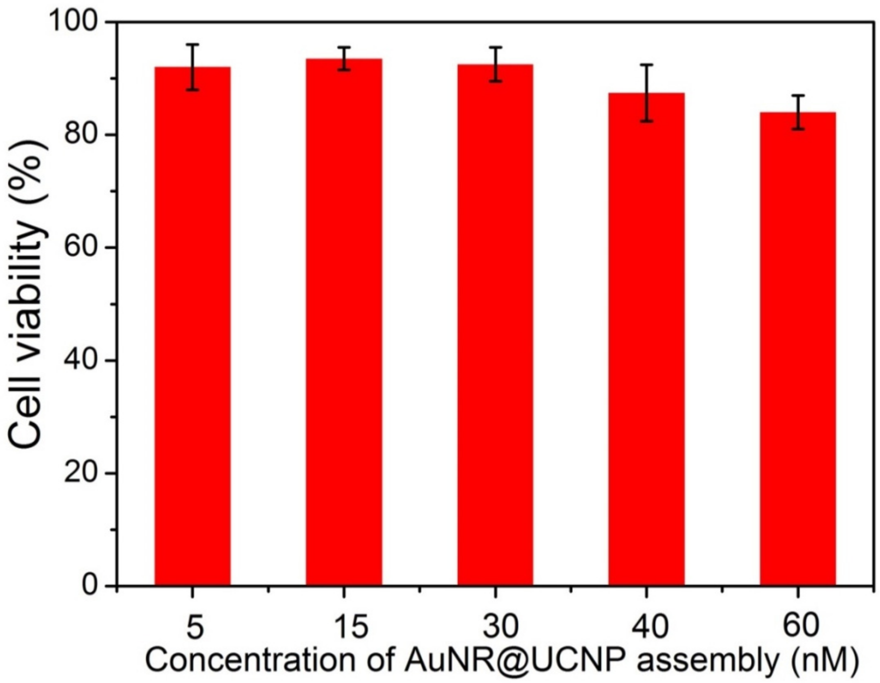

Fig. S15. Cell viabilities of Hela incubated with different concentrations (based on the concentration of AuNR) of AuNR@UCNP assembly without irradiation. The data are presented as the mean ± s.d. (n = 3).

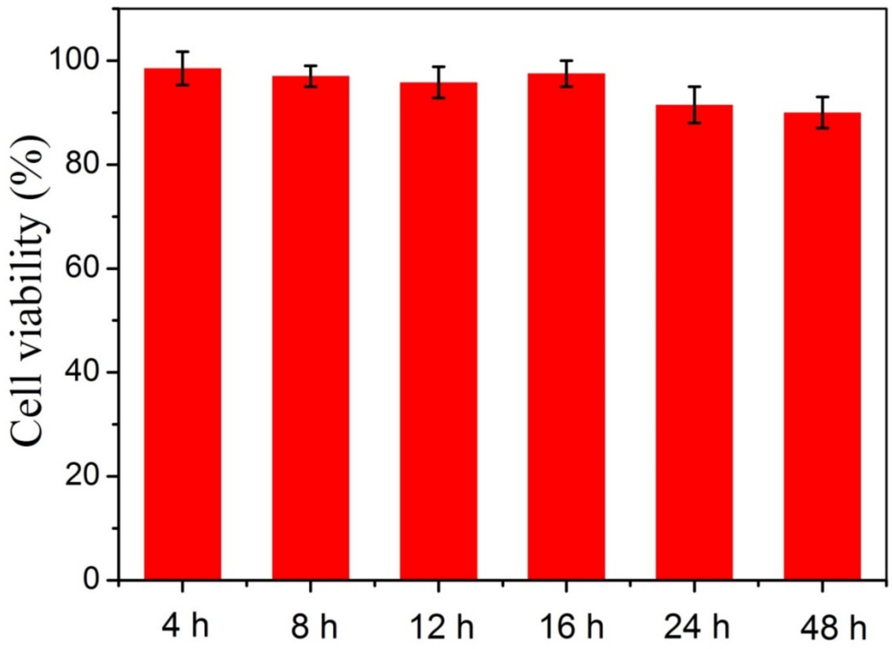

Fig. S16. Cell viabilities of Hela incubated with 30 nM AuNR@UCNP assembly for different times without irradiation. The data are presented as the mean ± s.d. (n = 3).

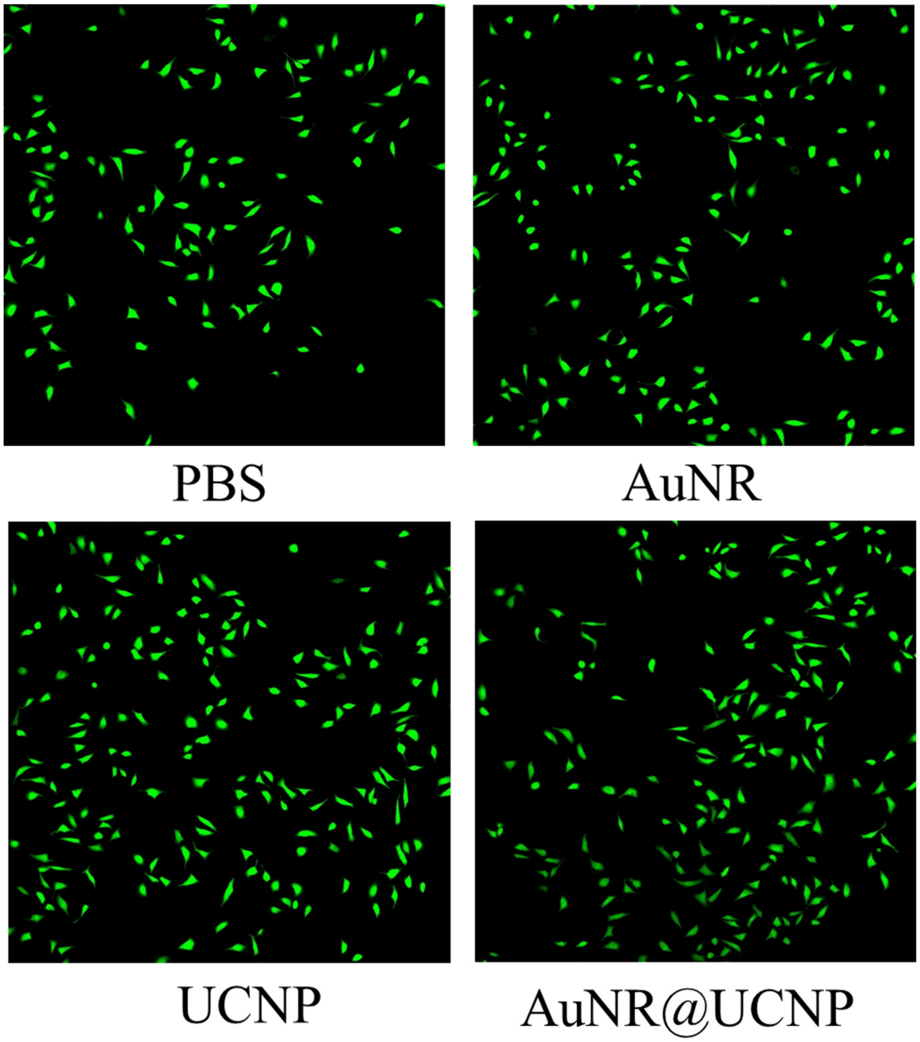

Fig. S17. Confocal fluorescence microscope image of live/dead assay co-stained HeLa cells after incubation with PBS, AuNR, UCNP and AuNR@UCNP, with 488nm and 552 nm laser irradiation, and the power is 200 mW.

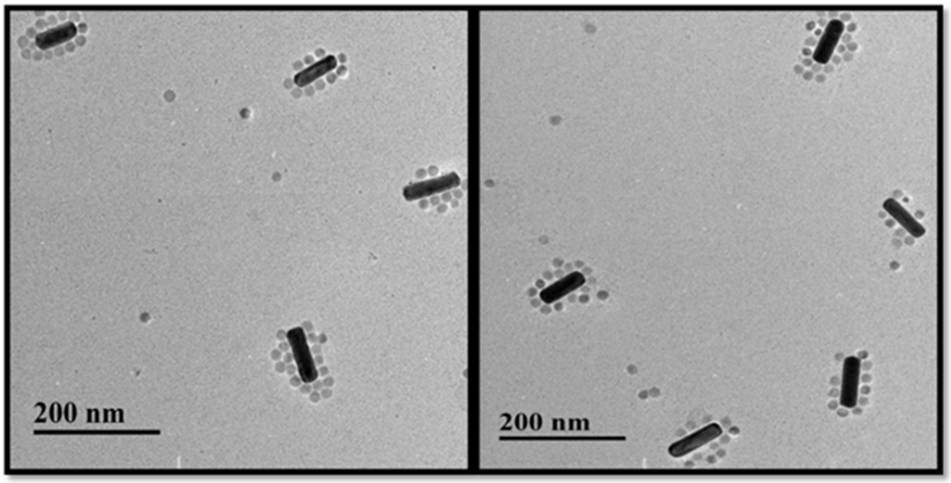

Fig. S18. TEM images of the AuNR@UCNP assembly after modification with TAT peptide.

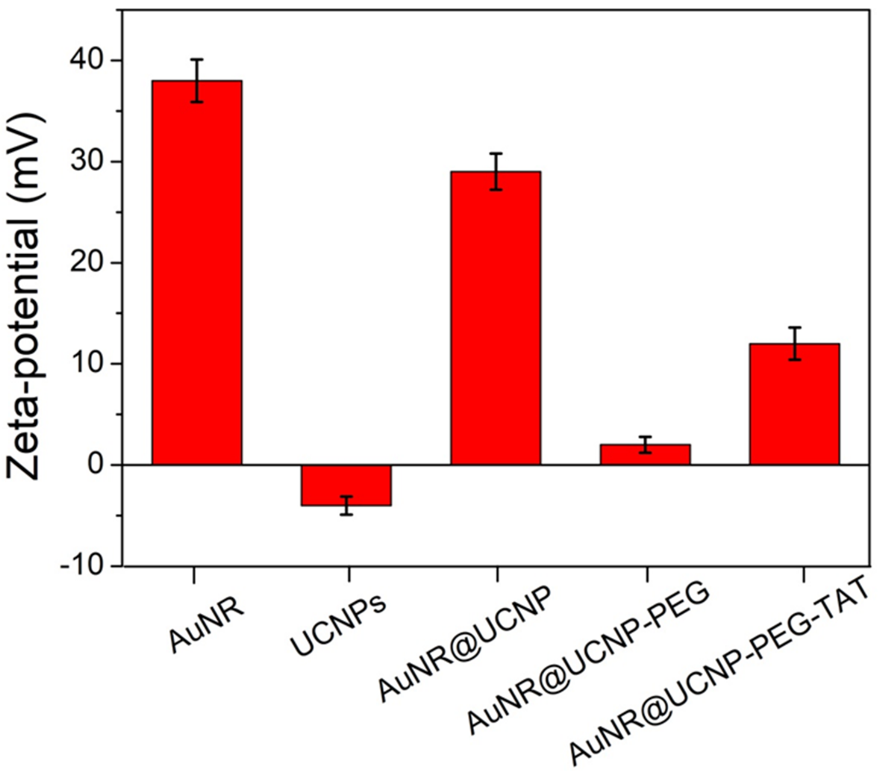

Fig. S19. Zeta potential of AuNR, UCNPs, AuNR@UCNP, AuNR@UCNP modified with PEG, and AuNR@UCNP modified with PEG and TAT.

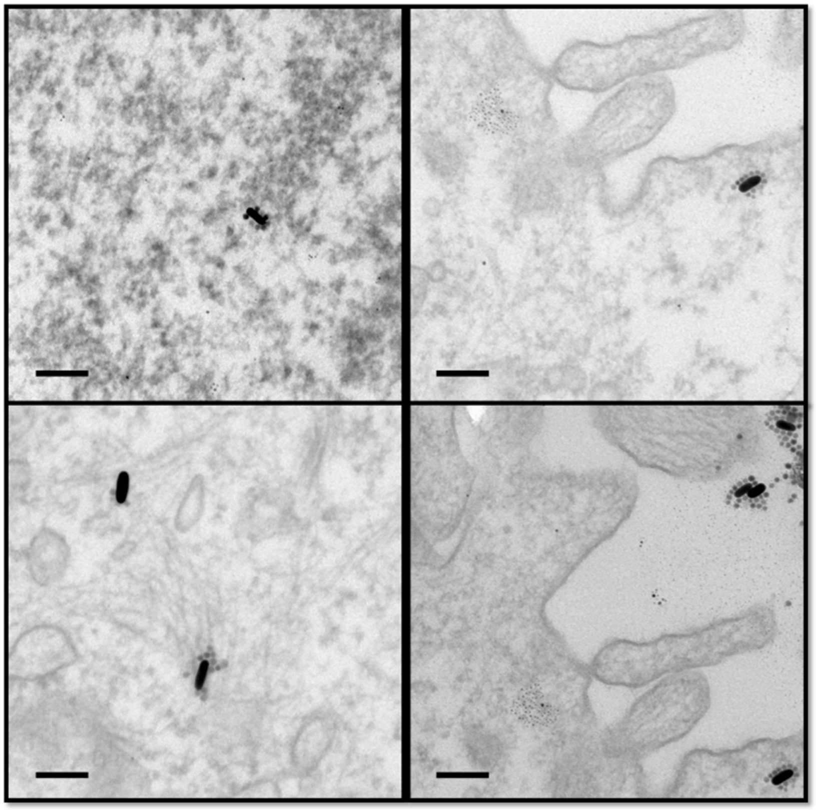

Fig. S20. Bio-TEM images of AuNR@UCNP assembly in Hela cells, scale bars 100 nm.

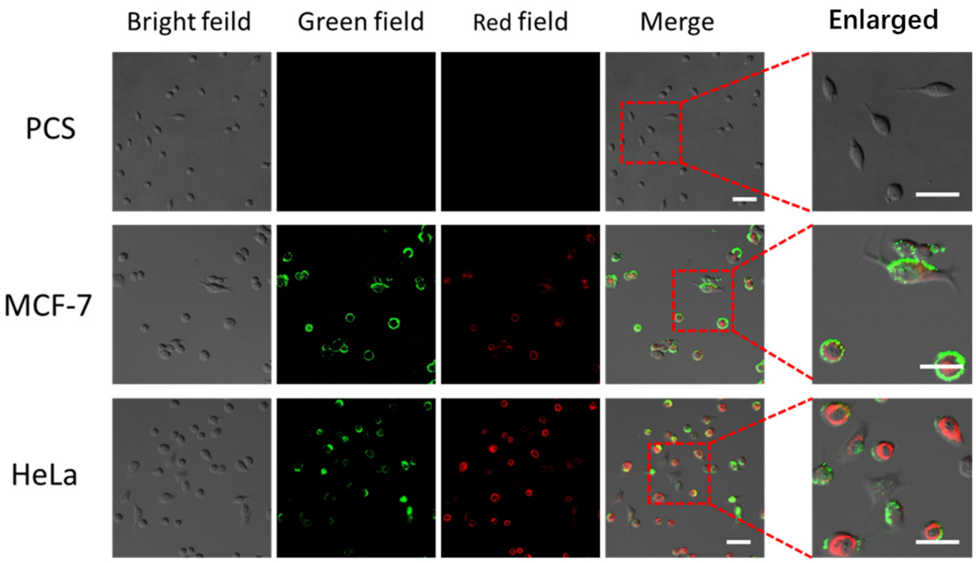

Fig. S21. Confocal images of PCS, MCF-7, HeLa cells with AuNR@UCNP assembly (980nm laser, 500 mW). Scale bars = 20 μm.

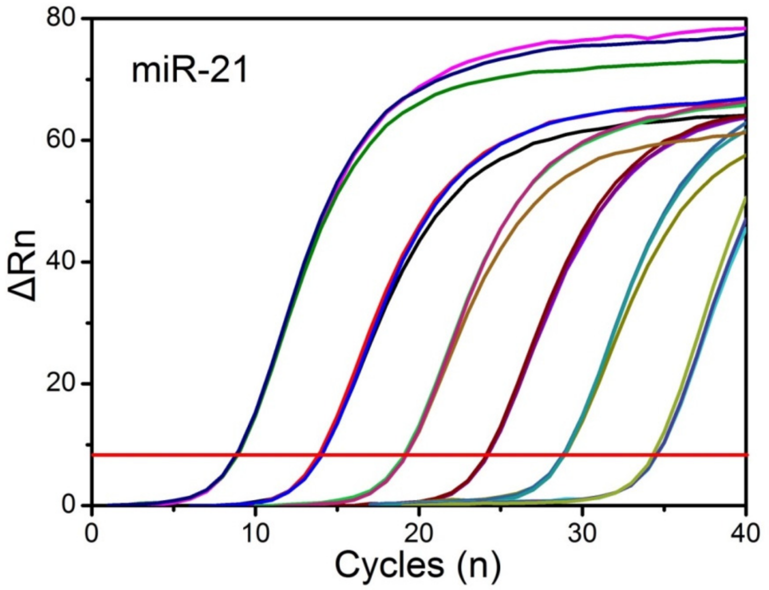

Fig. S22. Dynamic range and sensitivity of the TaqMan miR assay. Amplification plot of synthetic miR-21 over six orders of magnitude. Synthetic miR-21 input ranged from 200 aM to 2×10^7 aM in PCR.

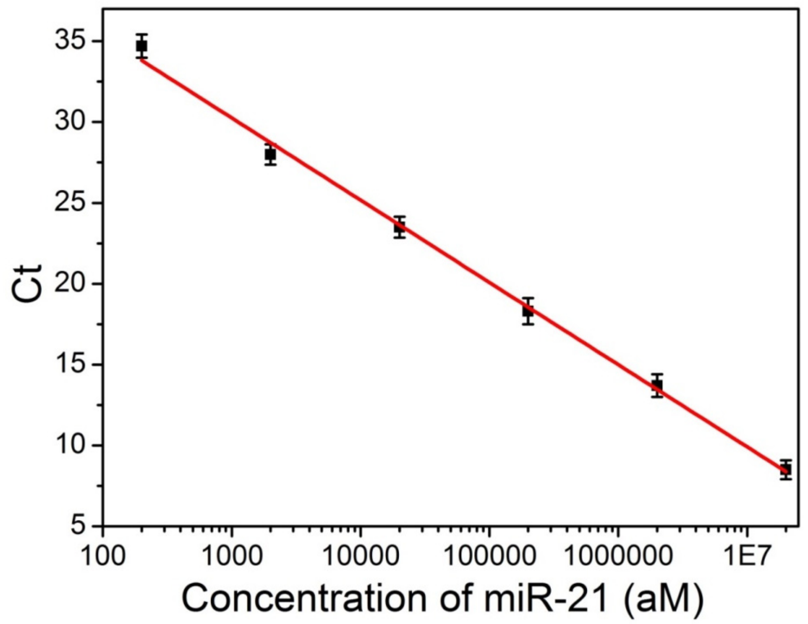

Fig. S23. Standard curve of the miR-21 in PCR. The data are presented as the mean ± s.d. (n = 3).

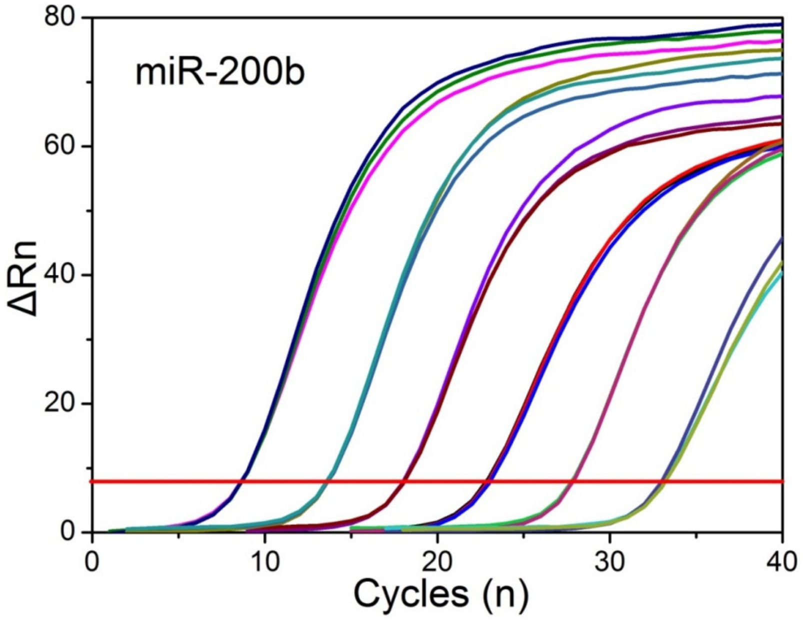

Fig. S24. Dynamic range and sensitivity of the TaqMan miR assay. Amplification plot of synthetic miR-200b over six orders of magnitude. Synthetic miR-21 input ranged from 100 aM to 1 × 10^7 aM in PCR.

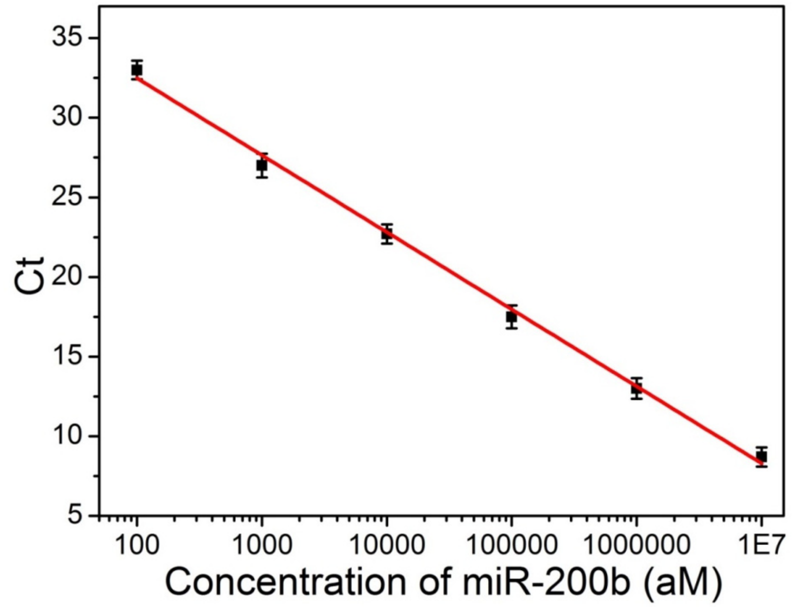

Fig. S25. Standard curve of the miR-200b in PCR. The data are presented as the mean ± s.d. (n = 3).

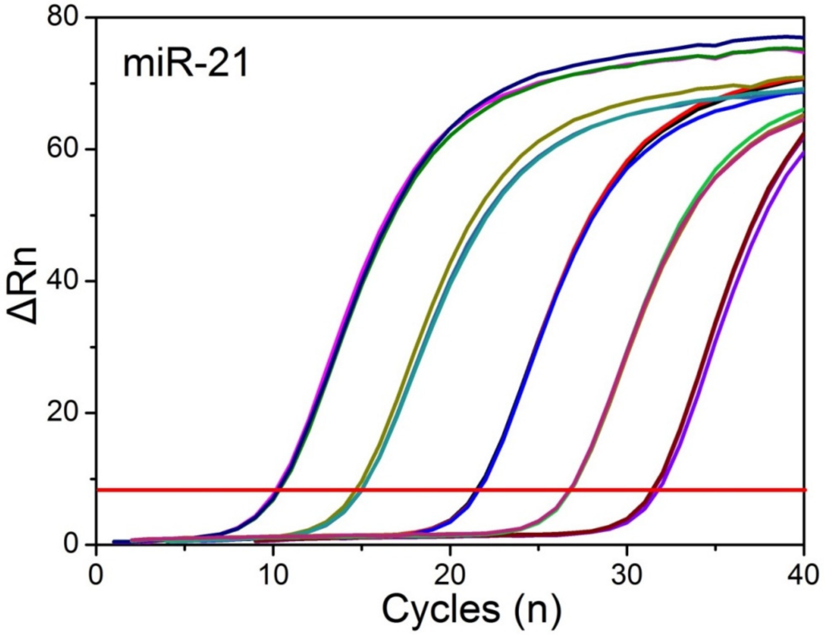

Fig. S26. qRT-PCR quantification of miR-21in transfected HeLa cells.

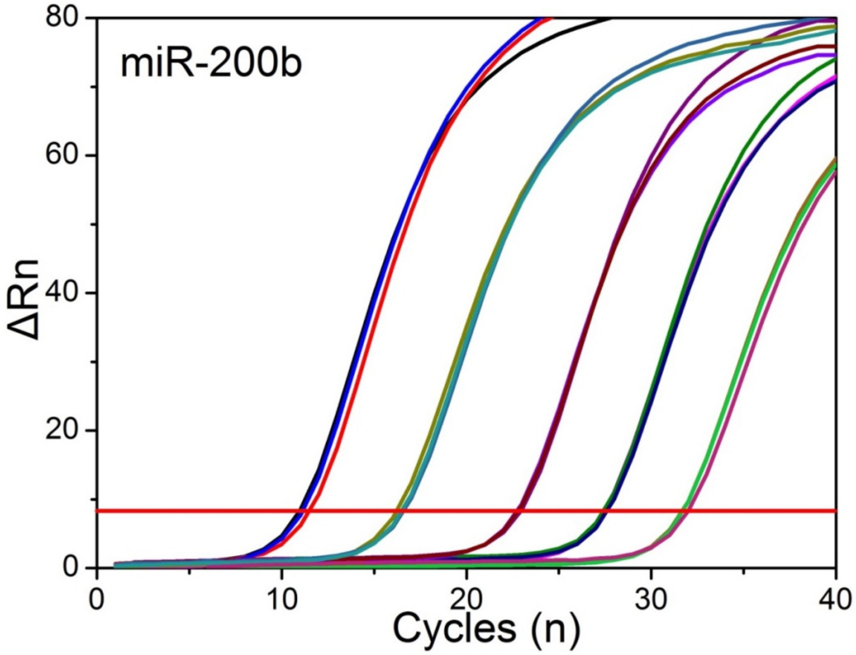

Fig. S27. qRT-PCR quantification of miR-200b in transfected HeLa cells.

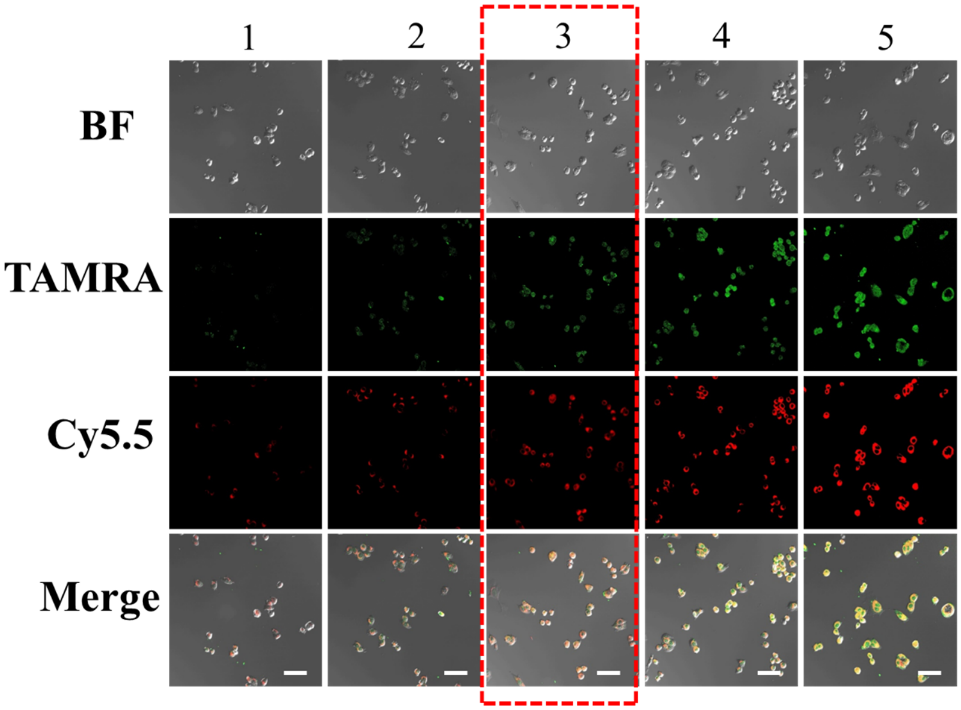

Fig. S28. Confocal images of HeLa cells with different amounts of miR-21 and miR-200b: columns 1 and 2 were transfected with antisense sequences of both miR-21 and miR-200b; column 3 was not transfected; columns 4 and 5 were transfected with both miR-21 and miR-200b. A continuous-wave near-infrared laser operating at 980 nm provided the excitation and the power is 500 mW, the images were collected at 588 ± 20 nm (green channel) and 736 ± 50 nm (red channel) (980nm laser, 500 mW). Scale bars are 20 μm.

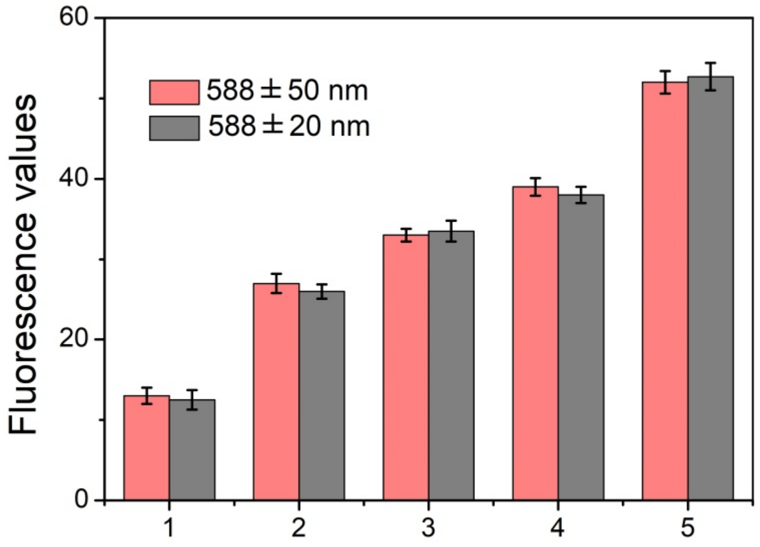

Fig. S29. Fluorescence intensity values acquired at 588 ± 50 nm and 588 ± 20 nm of HeLa cells with different amounts of miR-21 and miR-200b: columns 1 and 2 were transfected with antisense sequences of both miR-21 and miR-200b; column 3 was not transfected; columns 4 and 5 were transfected with both miR-21 and miR-200b. The data are presented as the mean ± s.d. (n = 3).

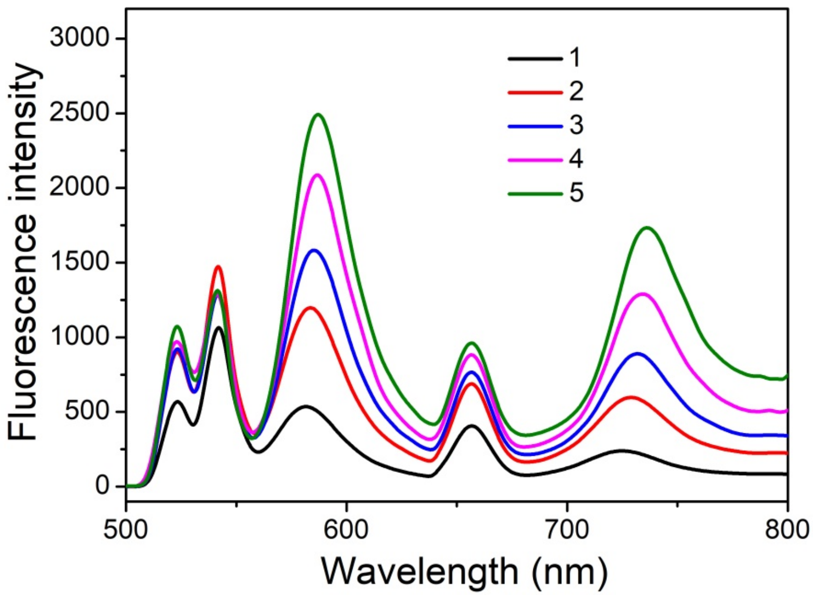

Fig. S30. Fluorescence spectra of different concentration of miR-21 and miR-200b Hela cells (10^6 cells/sample) after incubated with AuNR@UCNP assembly (980 nm laser, 500 mW). 1:0.032 amol/ngRNA miR-21 and 0.018 amol/ngRNA miR-200b; 2:0.21 amol/ngRNA miR-21 and 0.097 amol/ngRNA miR-200b; 3:0.38 amol/ngRNA miR-21 and 0.21 amol/ngRNA miR-200b; 4:0.83 amol/ngRNA miR-21 and 0.45 amol/ngRNA miR-200b; 5:2.97 amol/ngRNA miR-21 and 1.87 amol/ngRNA miR-200b.

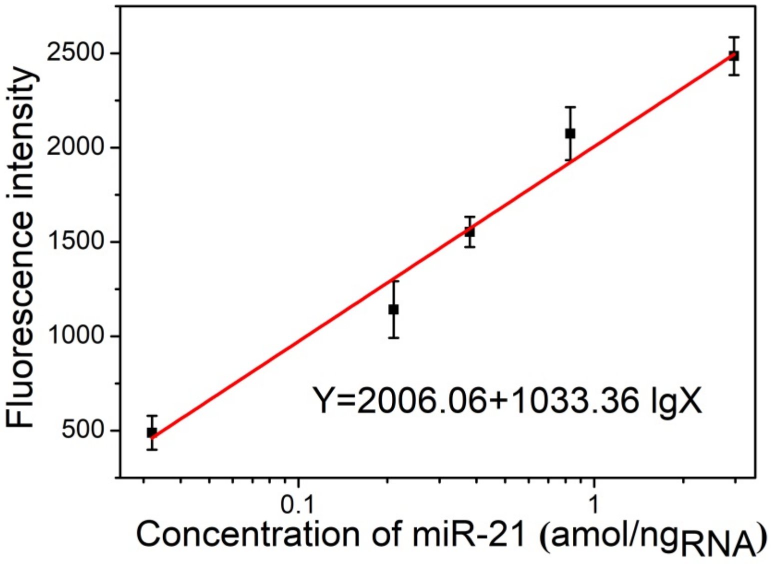

Fig. S31. Linear dependence of fluorescence intensity of TAMRA vs. different concentrations of miR-21 in Hela cells. The data are presented as the mean ± s.d. (n = 3).

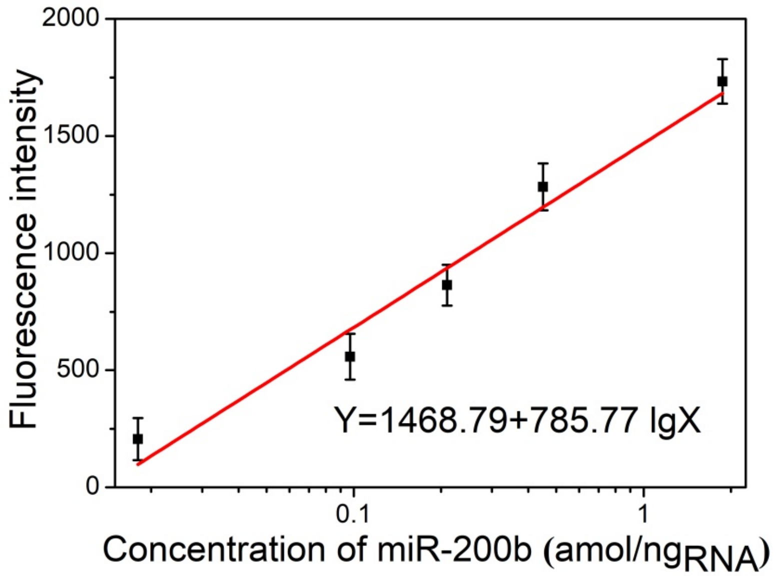

Fig. S32. Linear dependence of fluorescence intensity of Cy5.5 vs. different concentrations of miR-200b in Hela cells. The data are presented as the mean ± s.d. (n = 3).

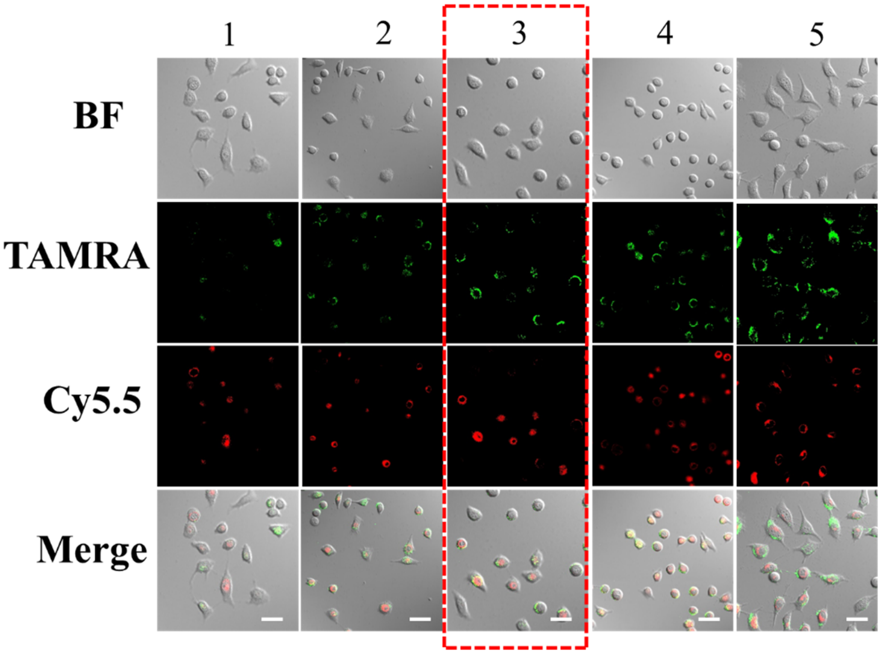

Fig. S33. Confocal images of HeLa cells with different amounts of miR-21 (1 and 2 were transfected with antisense sequences of miR-21; 3 was not transfected; 4 and 5 were transfected with miR-21, respectively) with assembly (980nm laser, 500 mW). Scale bars = 20 μm.

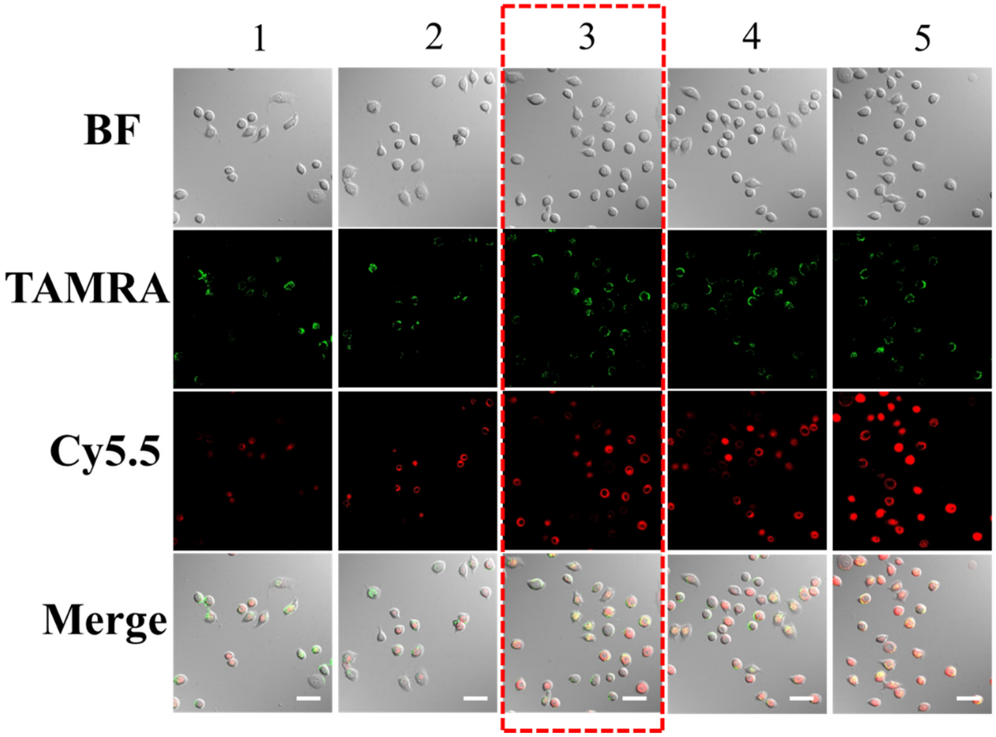

Fig. S34. Confocal images of HeLa cells with different amounts of miR-200b (1 and 2 were transfected with antisense sequences of miR-200b; 3 was not transfected; 4 and 5 were transfected with miR-21, respectively) with assembly (980 nm laser, 500 mW). Scale bars = 20 μm.

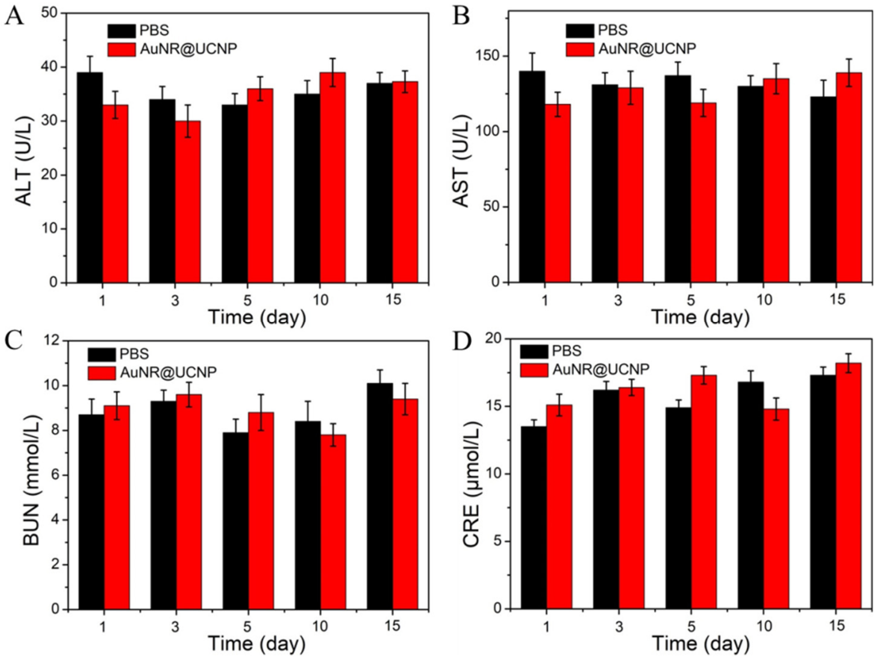

Fig. S35. Serum biochemistry of nude mice after AuNR@UCNP treatments. ALT, alanine aminotransferase; AST, aspartate aminotransferase; BUN, blood urine nitrogen; CRE, creatinine. The data are presented as the mean ± s.d. (n = 3). The data are presented as the mean ± s.d. (n = 3).

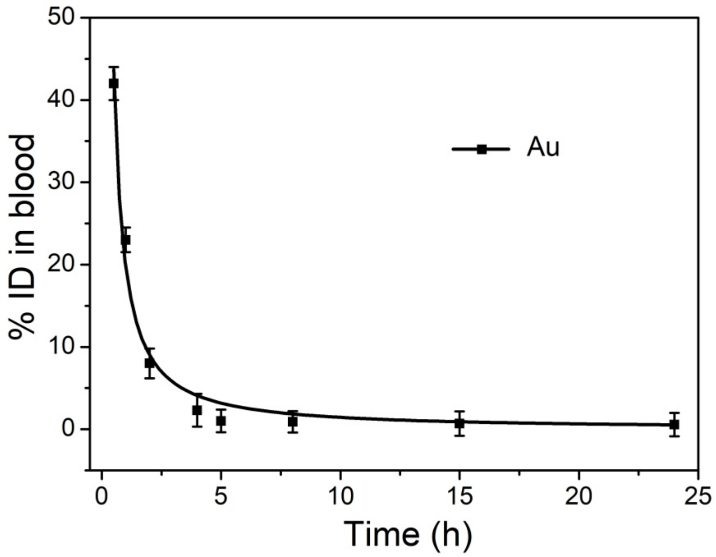

Fig. S36. Pharmacokinetic curves of AuNR@UCNP as analyzing the Au content in the blood by using ICP-MS, shown as the percentage of the injected dose (% ID). The data are presented as the mean ± s.d. (n = 3).

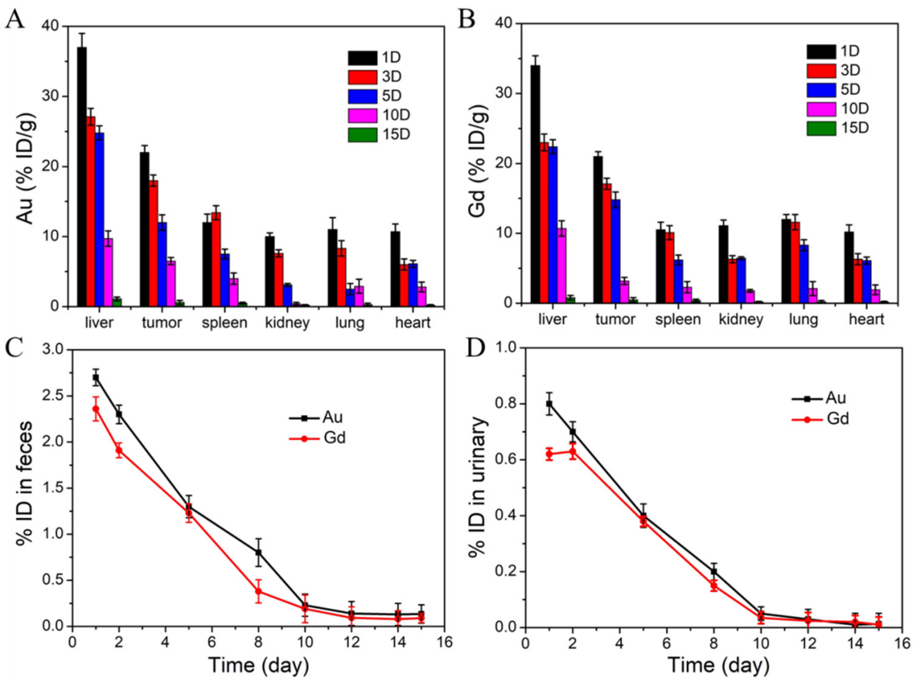

Fig. S37. The bio-distribution of AuNR@UCNP as analyzing the (A) Au and (B) Gd content using ICP-MS at different time points post-injection, expressed as the percentage of the injected dose per gram of tissue (% ID/g); In vivo excretion of AuNR@UCNP as analysed by the Au and Gd content in (C) fecal excretion and (D) urinary excretion at different time points. The data are presented as the mean ± s.d. (n = 3).

Table S1. DNA or RNA sequences used in NP assembly and miRs detection.

Name	Sequence (5' to 3')	Modification
DNA_1	AAAAATCATCTATCAACATCAGTCTGATAAGCTATAGAAGC	5' SH
DNA_2	AAAAAGCTTGA	5' SH
DNA_3	AAAGCTTC	5' TAMRA
DNA_4	AAAAATCATCTATCATCATTACCAGGCAGTATTAAACGTCG	5' SH
DNA_5	AAAAAATATGA	5' SH
DNA_6	AAACGACG	5' Cy5.5
normal DNA_1	AAAAATCATCTATCAACATCAGTCTGATAAGCTATAGAAGC	5' SH
normal DNA_2	AAAAAGCTTGA	5' SH
normal DNA_3	AAAGCTTC	5' TAMRA
normal DNA_4	AAAAATCATCTATCATCATTACCAGGCAGTATTAAACGTCG	5' SH
normal DNA_5	AAAAAATATGA	5' SH
normal DNA_6	AAACGACG	5' Cy5.5
miR-21	UAGCUUAUCAGACUGAUGUUGA	
anti-miR-21	UCAACAUCAGUCUGAUAAGCUA	
mismatched 1	UAACUUAUCAGACUGAUGUUGA	
miR-200b	UAAUACUGCCUGGUAAUGAUGA	
anti-miR-200b	UCAUCAUUACCAGGCAGUAUUA	
mismatched 2	UAGUACUGCCUGGUAAUGAUGA	
Let-7d	AGAGGUAGUAGGUUGCAUAGUU	
miR-203[b]	GUGAAAUGUUUAGGA CCA CUA G	

Table S2. Comparisons with other sensing systems of intracellular miR-21 detection.

Number	Detection Method	LOD of miR-21	Ref.
1	gold-quantum dot core–satellite	0.05 amol/ng RNA	[4]
2	Ru(II) ECL system	3.3 aM	[5]
3	DNA-gold nanoparticle probes	0.02 amol/ng RNA	[6]
4	hybridization chain reaction	6.8×10^{6} aM	[7]
5	Hybrid Nanoparticle Pyramids	0.23 fmol/ 10μgRNA	[8]
6	three-mode electrochemical sensing	5 aM	[9]
7	Quadratic isothermal amplification	1 aM	[10]
8	label-free electrochemical biosensor	8 aM	[11]
9	Au NR dimer-UCNP core–satellite (CS) nanostructures based on SERS	0.011 amol/ng_{RNA}	[12]
10	Au-UCNP pyramids luminescence detection	0.012 amol/ng RNA	[13]
11	AuNR-Pt@Ag_2S core–satellite	0.0082 amol/ng RNA	[14]
12	This work	3.2 zmol/ng RNA	

Supplementary References

(1) Liu, C.; Gao, Z.; Zeng, J.; Hou, Y.; Fang, F.; Li, Y.; Qiao, R.; Shen, L.; Lei, H.; Yang, W.; Gao, M. *ACS Nano* **2013**, *7* (8), 7227.

(2) Krpetic, Z.; Saleemi, S.; Prior, I. A.; See, V.; Qureshi, R.; Brust, M. *ACS Nano* **2011**, *5* (6), 5195-5201.

(3) Sun, M. Z.; Xu, L. G.; Banhg, J. H.; Kuang, H.; Alben, S.; Kotov, N. A.; Xu, C. L. Nat. Commun. **2017**, 8, 1847.

(4) Zhao, X.; Xu, L.; Sun, M.; Ma, W.; Wu, X.; Kuang, H.; Wang, L.; Xu, C. *Small* **2016**, *12* (34), 4662.

(5) Chen, A.; Gui, G.-F.; Zhuo, Y.; Chai, Y.-Q.; Xiang, Y.; Yuan, R. *Anal. Chem.* **2015**, *87* (32), 6328.

(6) Degliangeli, F.; Kshirsagar, P.; Brunetti, V.; Pompa, P. P.; Fiammengo, R. *J. Am. Chem. Soc.* **2014**, *136* (6), 2264.

(7) Miao, X.; Ning, X.; Li, Z.; Cheng, Z. *Sci. Rep.* **2016**, *6*, 32358.

(8) Li, S.; Xu, L.; Sun, M.; Wu, X.; Liu, L.; Kuang, H.; Xu, C. *Adv. Mater.* **2017**, *29* (19), 1606086.

(9) Labib, M.; Khan, N.; Ghobadloo, S. M.; Cheng, J.; Pezacki, J. P.; Berezovski, M. V. *J. Am. Chem. Soc.* **2013**, *135* (8), 3027.

(10) Duan, R. X.; Zuo, X. L.; Wang, S. T.; Quan, X. Y.; Chen, D. L.; Chen, Z. F.; Jiang, L.; Fan, C. H.; Xia, F. *J. Am. Chem. Soc.* **2013**, *135* (12), 4604.

(11) Zhang, X.; Wu, D. Z.; Liu, Z. J.; Cai, S. X.; Zhao, Y. P.; Chen, M.; Xia, Y. K.; Li, C. Y.; Zhang, J.; Chen, J. H. *Chem. Commun.* **2014**, *50* (82), 12375.

(12) Ma, W.; Fu, P.; Sun, M.; Xu, L.; Kuang, H.; Xu, C. *J. Am. Chem. Soc.* **2017**, *139* (34), 11752.

(13) Li, S.; Xu, L.; Ma, W.; Wu, X.; Sun, M.; Kuang, H.; Wang, L.; Kotov, N. A.; Xu, C. *J. Am. Chem. Soc.* **2016**, *138* (1), 306.

(14) Qu, A.; Xu, L.; Sun, M.; Liu, L.; Kuang, H.; Xu, C. *Adv. Funct. Mater.* **2017**, *27* (46), 1703408.
